# Metaproteomic profiling of fungal gut colonization in gnotobiotic mice

**DOI:** 10.1186/s42523-022-00163-2

**Published:** 2022-02-22

**Authors:** Veronika Kuchařová Pettersen, Antoine Dufour, Marie-Claire Arrieta

**Affiliations:** 1 Department of Physiology and Pharmacology, Health Research Innovation Centre, University of Calgary, 3330 Hospital Drive N.W., Calgary, AB T2N 4N1 Canada; 2 Department of Pediatrics, University of Calgary, Calgary, Canada; 3 International Microbiome Centre, Cumming School of Medicine, University of Calgary, Calgary, Canada; 4 Department of Medical Biology, UiT The Arctic University of Norway, Tromsø, Norway

**Keywords:** Early life gut microbiome, Quantitative Proteomics, Metaproteomics, Gut fungi, Gnotobiotic murine model, Antimicrobials

## Abstract

**Background:**

Eukaryotic microbes can modulate mammalian host health and disease states, yet the molecular contribution of gut fungi remains nascent. We previously showed that mice exclusively colonised with fungi displayed increased sensitivity to allergic airway inflammation and had fecal metabolite profiles similar to germ-free mice. This marginal effect on the host metabolome suggested that fungi do not primarily use metabolites to modulate the host immune system.

**Methods:**

To describe functional changes attributed to fungal colonisation, we performed mass spectrometry-based analyses of feces (Label-Free Quantitative; LFQ) and the small intestine (labeling with Tandem Mass Tag; TMT) of gnotobiotic mice colonised with defined consortia of twelve bacterial species, five fungal species, or both. We also evaluated the effect of microbiome perturbances on the metaproteome by analysing feces from mouse pups treated with an antibiotic or antifungal.

**Results:**

We detected 6675 proteins in the mice feces, of which 3845 had determined LFQ levels. Analysis of variance showed changes in the different gnotobiotic mouse groups; specifically, 46% of 2860 bacterial, 15% of 580 fungal, and 76% of 405 mouse quantified proteins displayed differential levels. The antimicrobial treatments resulted in lasting changes in the bacterial and fungal proteomes, suggesting that the antimicrobials impacted the entire community. Fungal colonisation resulted in changes in host proteins functional in innate immunity as well as metabolism, predicting specific roles of gut fungi on host systems during early developmental stages. Several of the detected fungal proteins (3% of 1492) have been previously reported as part of extracellular vesicles and having immunomodulating properties. Using an isobaric labelling TMT approach for profiling low abundant proteins of the jejunal tissue, we confirmed that the five fungal species differentially impacted the host intestinal proteome compared to the bacterial consortium. The detected changes in mouse jejunal proteins (4% of 1514) were mainly driven by metabolic proteins.

**Conclusions:**

We used quantitative proteomic profiling of gnotobiotic conditions to show how colonisation with selected fungal species impacts the host gut proteome. Our results suggest that an increased abundance of certain gut fungal species in early life may affect the developing intracellular attributes of epithelial and immune cells.

**Supplementary Information:**

The online version contains supplementary material available at 10.1186/s42523-022-00163-2.

## Introduction

The fungal portion of the mammalian gut microbiome, also referred to as the mycobiome, is estimated to constitute less than 0.1% of the gut ecosystem [1, 2]. Although it has been significantly less characterised than the bacterial microbiome, the mycobiome has an important and often overlooked role in host health and disease [3–5]. Cell numbers alone do not provide a comparable measure for the microbiome communities as fungal cells are up to 100 times bigger in volume and with genomes from 4 to 200 times larger than most bacteria [6], representing large biomass with potent production capacities for proteins and metabolites. Community ecology provides many examples of low abundance species with sizable impacts on community structure and function, showing that species’ importance cannot be predicted based upon their abundance in an ecosystem [7–9].

Fungi are an essential part of terrestrial and aquatic ecosystems, engaging in a wide variety of relationships with other members of microbial communities (*i.e.*, bacteria, archaea, viruses) and their plant and animal hosts [10–12]. Bacteria modulate fungi’s ability to colonise mammalian hosts, as evidenced by clinical [13] and murine [14, 15] studies describing the effects of antibiotic treatment on the gut mycobiome. In general, antibiotic use promotes fungal overgrowth, and its immunological consequences can be detected at distant sites such as the lung [13, 15]. Similarly, antifungals affect bacterial community structure and can have adverse effects on the host health [16]. Controlled release of inbred laboratory mice into an outdoor enclosure further demonstrated the impact of fungi on the host immune functions [5]. Although the mice rewilding enhanced the differentiation of immune cell populations previously associated with pathogen exposure, the resulting fungi-induced immune system changes occurred in the absence of an infection.

The inter- and intra-kingdom relations between microbes of the gut microbiome have been mostly described using the relative species abundance derived by DNA sequencing methods [17]. However, this approach does not characterise molecular interactions between microbes that occur directly through secreted molecules and by physical contact, or indirectly by modulating the host immune response. Using a gnotobiotic mice model, we previously demonstrated the impact of gut fungal colonisation on a defined bacterial consortium and the host [18]. Gut fungi promoted shifts in bacterial microbiome ecology, and mice colonised exclusively with fungi displayed immunological features related to atopy in early life. Intriguingly, gut fungi had a marginal effect on the host fecal metabolome [18], suggesting that other functional mechanisms are at play. The results led us to investigate the host-microbiome interactions at the protein level, taking advantage of the defined experimental conditions, the availability of the bacterial species’ genomes, and reference protein databases for the fungal species and mouse host.

**Fig. 1:**
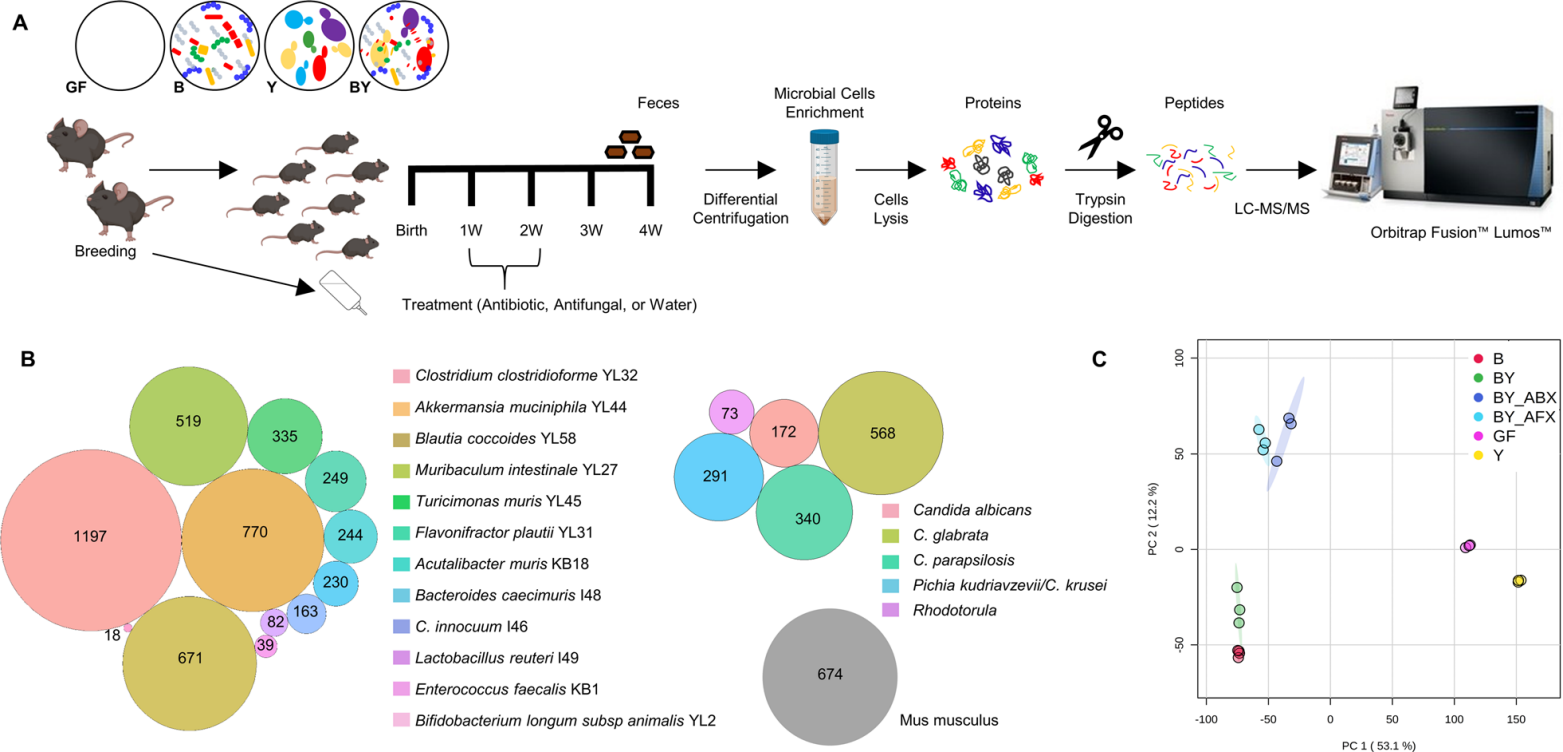
Description of a gnotobiotic colonisation model by label-free quantitative metaproteomics. **A** Experimental design of shotgun label-free proteomic experiment, including enrichment of fecal microbial cells by differential centrifugation. **B** Detected fecal metaproteome composed of proteins originating from 12 bacterial, 5 fungal species, and mouse. **C** PCA score plot based on the relative levels of 3845 proteins quantified for mouse, bacteria, and fungi/yeast. X- and Y-axis show the first and second principal components, accounting for 53.1% and 12.2% of the total variation, respectively. Abbreviations of the mice treatment groups: B, bacteria; BY, bacteria-yeast; BY_ABX/AFX, bacteria-yeast and antibiotic or antifungal treatment; GF, germ-free; Y, fungi/yeast

Here, we show proteomic analyses of feces and small intestine of gnotobiotic mice, which were colonised with either 12 bacterial species (Bacteria, -B), a group of 5 fungal/yeast species (Yeast, -Y), or a combination of the 17 fungi and bacteria (Bacteria and Yeast, -BY) (Fig. 1A). We utilised the Oligo-MM^12^ bacterial consortium of mouse-derived strains that are persistent, inheritable and elicit an immune response in mice similar to a complex microbiota [19, 20]. For fungi, we selected six fungal strains from taxa that commonly colonise the human gut [21, 22] and have been previously linked to atopy and asthma risk [23, 24]. We also evaluated the effect of microbiome perturbances to the metaproteome by analysing feces from mouse pups treated with antibiotic Augmentin (BY_ABX) or antifungal Fluconazole (BY_AFX). We selected Augmentin (Amoxicillin/Clavulanic acid) a broad-spectrum antibiotic commonly used in the paediatric population [25], as the animal model was designed to mimic adverse factors affecting the early life gut microbiome.

We observed dynamic relationships between the bacteria and fungi that were reflected by changes in the individual species proteome profiles derived from different colonisation and treatment conditions. Metaproteomic analyses based on label-free quantification (LFQ) of fecal proteins documented an extensive impact of microbial colonisation on the host and revealed new features of the host protein response to fungal colonisation. Proteomics of the jejunal tissue based on labeling with Tandem Mass Tag (TMT) provided further insights on the host cellular pathways impacted by the microbial colonisation and confirmed that gut fungi elicit distinct effects compared to bacteria.

## Materials and methods

### Gnotobiotic mice

Germ-free (GF) C57Bl/6J mice were obtained from and housed at the gnotobiotic mouse facility of the International Microbiome Centre (IMC) at the University of Calgary. Details of the animal experiments have been previously described [18], and all the animal work was conducted following animal protocols approved by the Institutional Animal Care and Use Committee. Briefly, female adult GF mice were orally gavaged twice, three days apart, with 100 μl of a consortium of microorganisms or kept under germ-free conditions. Colonisation consisted of consortia of (i) 12 mouse-derived bacteria [19] (B—bacteria), (ii) five yeast species previously linked to atopy and asthma risk in infants [26, 27] (Y—Yeast), or (iii) a combination of all 17 bacterial and yeast species (BY) (Fig. 1A, Additional file 2: Table S1). We used previously described method for mice colonisation with the Oligo-MM^12^ consortium [19], in which inocula were prepared under anaerobic conditions by mixing 100 μl of 2-day-old microbial cultures of each species. Bacteria were grown in fastidious anaerobe broth (LabM, Heywood, England, United Kingdom), and yeasts were grown in yeast-mold broth (YM; BD, Sparks, Maryland, USA). After the second gavage, mice were paired for mating on a 2:1 female:male ratio per cage. Two breeding pairs were used for each group, and they produced on average 9 offspring (± 2) per colonisation condition, which constituted individual biological replicates in the gnotobiotic experiment. To ensure microbial colonisation with the desired consortia in the offspring, the corresponding inocula were further spread on the dams abdominal and nipple regions on days 3 and 5 after birth [28]. Microbial engraftment was confirmed by sequencing [18, 29], with an intestinal abundance of bacterial communities resembling previously described composition of the Oligo-MM^12^ consortium [19]. Two groups of mice colonised with both bacteria and yeasts were treated with the antibiotic Augmentin (0.2 mg/ml; MilliporeSigma, St. Louis, Missouri, USA) or antifungal Fluconazole (0.5 mg/ml; MilliporeSigma) in sterile drinking water from day 7 to 14 after birth (groups BY_ABX and BY_AFX, respectively). Treatment solutions were prepared by dissolving the antimicrobials in distilled water, followed by filter sterilisation. Mice were kept at maximum five animals per isolator cage and housed inside gnotobiotic flexible-film isolators, under a 12-h light/12-h dark cycle, 40% relative humidity, 22–25 °C, and ad libitum access to sterile food and water.

### Enrichment of microbial cells from fecal sample

Fecal samples were collected from gnotobiotic mice at the end of their 3^rd^ week of life and immediately stored at − 80 °C until use. After thawing at 4 °C, pooled samples of ~ 300 mg, originating from co-housed mice of the same treatment group, were subjected to differential centrifugation to enrich for microbial cells, according to previously described methodology [30]. Briefly, each sample was resuspended in 4 ml of Phosphate-Buffered Saline (PBS), homogenised by using GentleMACS C tubes (Miltenyi Biotec, Bergisch Gladbach, Germany), and subjected to low-speed centrifugation at 20×*g* for 5 min (Centrifuge 5810 R, Eppendorf, Hamburg, Germany) to eliminate gross particulate material. The supernatant was transferred to 50 ml conical centrifuge tube and kept at 4 °C, whereas the pellet was resuspended in PBS. The washing step was repeated until the supernatant appeared translucent (5–7 times). The collected supernatant was centrifuged at 3214×*g* for 1 h, and the resulting pellet was subjected to cell lysis and protein digestion described below. A quality control step comprising of microscopic examination of Gram-stained fractions of the pellet was included to confirm bacterial and fungal cell extraction.

### Murine intestinal tissue sample

Jejunal tissue dissected from the small intestine of 4-weeks old mice was cleaned of luminal debris with PBS and snap-frozen in liquid nitrogen and stored at − 80 °C until further processing.

### Protein extraction

Enriched fecal microbiota and jejunal tissue samples were resuspended in lysis buffer [2% sodium dodecyl sulfate, 100 mM Triethylammonium bicarbonate (TEAB) buffer, 10 mM Ethylenediaminetetraacetic acid, and 1X Complete Mini EDTA free protease inhibitors (F. Hoffmann-La Roche AG, Basel, Switzerland), pH 8.0] in 1:4 w/v ratio and transfer into a 2 ml screw-cap tube with FastPrep Lysing matrix A (MP Biomedicals, Irvine, California, USA). Cells were mechanically disrupted by bead-beating for 2 × 3 min at 30 Hz (TissueLyser II, Qiagen, Hilden, Germany). The samples were incubated at − 80 °C for 10 min and at 95 °C for 10 min, followed by centrifugation for 30 min at 18,000×*g*, 4 °C. To disrupt released cellular DNA that would interfere with downstream protein quantification, the supernatants were sonicated 3 × for 10 s with 20 s resting intervals on ice. Sonicated samples were centrifuged at 18,000×*g*, 4 °C, for 10 min, the supernatants collected, and protein concentration was measured by using DeNovix Spectrophotometer (DeNovix Inc., Wilmington, DE, USA).

### Sample preparation for label-free quantitative (LFQ) proteomics

The cell lysates of fecal microbiota-enriched samples were processed according to Filter-Aided Sample Preparation protocol [30, 31]. Briefly, cell lysates containing 500 μg of total protein were incubated with 10 mM DTT in 100 mM Ammonium Bicarbonate (AmBic) at the solution to total protein ratio (v/w) 1:10 for 45 min at 56 °C. The samples were then mixed with 8 M Urea in 10 mM HEPES pH 8.0 (UA) in YM-30 Microcon filter units (MilliporeSigma) and centrifuged at 10,000×*g* for 15 min. After discarding the eluate, the filtration units were washed once with the UA buffer (10,000×*g*, 15 min). Next, 50 mM iodoacetamide in UA was added to the filter, and samples were incubated in the dark for 20 min. The filter was washed twice with UA, followed by two washes with 50 mM AmBic. Proteins were digested with trypsin (Promega, Madison, Wisconsin, USA) in 40 mM AmBic at 37 °C for 18 h (enzyme to protein ratio (v/w) of 1:100). The resulting peptide mixtures were desalted by using SepPak C18 solid-phase extraction cartridges (Waters, Mississauga, Ontario, Canada), lyophilised at 30 °C in a vacuum concentrator (Speed Vac Plus SC110, Savant Instruments Inc., Holbrook, New York, USA) and stored at − 80 °C until further analysis. Prior to the LC–MS/MS analysis, the peptide mixtures were resuspended in 1% formic acid (FA). An aliquot of the tryptic digests was used to determine the concentration of the peptide mixtures by using Pierce™ Quantitative Colorimetric Peptide Assay (Thermo Fisher Scientific, Waltham, Massachusetts, USA).

### Tandem mass tag (TMT) labelling

TMT samples were prepared according to the manufacturer’s instructions (TMTsixplex™ Isobaric Label Reagent Set, Thermo Fisher Scientific). Briefly, 150 μg of jejunal tissue cell lysates were reduced by incubating with 200 mM Tris (2-carboxyethyl) phosphine hydrochloride (TCEP) at 55 °C for 1 h and alkylated with 375 mM iodoacetamide in the dark for 30 min. The proteins were precipitated by acetone, resuspended in 50 mM TEAB, and digested with trypsin at 37 °C for 18 h. Resulting peptide mixtures originating from 4 treatment groups (GF, B, BY, Y) and 5 biological replicates (n = 5) were labelled with TMT reagents distributed into 4 experimental groups (Additional file 1: Figure S1). Each experiment had one TMT tag (131) that contained pooled samples, created by combining equal amounts of peptides (20 ug) of each sample from one experimental group. The pooled samples served as internal standards for normalising the data across the experimental setup. For labelling, the peptides were incubated with TMT reagents for 1 h at room temperature. The reaction was quenched by adding 5% hydroxylamine, followed by incubation for 15 min. The resulting four multiplexed samples were desalted and quantified as described above.

### Mass spectrometry data acquisition

Liquid Chromatography: Tryptic peptides were analysed on an Orbitrap Fusion Lumos Tribrid mass spectrometer (Thermo Fisher Scientific) operated with Xcalibur (version 4.0.21.10) and coupled to a Thermo Scientific Easy-nLC (nanoflow Liquid Chromatography) 1200 system. Tryptic peptides (2 μg) were loaded onto a C18 trap (75 μm × 2 cm; Acclaim PepMap 100, P/N 164946; Thermo Fisher Scientific) at a flow rate of 2 μl/min of solvent A (0.1% FA and 3% acetonitrile in LC–MS grade water). Peptides were eluted using a 120 min gradient from 5 to 40% (5% to 28% in 105 min followed by an increase to 40% B in 15 min) of solvent B (0.1% FA in 80% LC–MS grade acetonitrile) at a flow rate of 0.3 μL/min and separated on a C18 analytical column (75 μm × 50 cm; PepMap RSLC C18; P/N ES803; Thermo Fisher Scientific). The Orbitrap Lumos was calibrated before each batch and 100 fmol of Pierce BSA protein digest (PI88341) was injected to control the performance of the LC and the mass spectrometer before the samples were acquired. The dates of sample runs are shown in Additional file 2: Table S1.

LFQ: Peptides were electrosprayed using 2.1 kV voltage into the ion transfer tube (300 °C) of the Orbitrap Lumos operating in positive mode. The Orbitrap first performed a full MS scan at a resolution of 120,000 FWHM to detect the precursor ion having m/z between 375 and 1575 and a + 2 to + 7 charge. The Orbitrap automatic gain control (AGC) and the maximum injection time were set at 4 × 10^5^ and 50 ms, respectively. The Orbitrap was operated using the top speed mode with a 3 s cycle time for precursor selection. The most intense precursor ions presenting a peptidic isotopic profile and having an intensity threshold of at least 5000 were isolated using the quadrupole and fragmented by higher-energy collisional dissociation (HCD, 30% collision energy) in the ion routing multipole. The fragment ions (MS2) were analysed in the ion trap at a rapid scan rate. AGC and the maximum injection time were set at 1 × 10^4^ and 35 ms, respectively, for the ion trap. Dynamic exclusion was enabled for 45 s to avoid the acquisition of the same precursor ion having a similar m/z (plus or minus 10 ppm).

MS3-TMT: Analysis of TMT labelled peptide mixtures was carried out on the Orbitrap Fusion Lumos Tribrid mass spectrometer (Thermo Scientific) (control software Xcalibur™, version 4.0.21.10) using a data-dependent method with multi-notch synchronous precursor selection MS3 scanning for TMT tags. The Orbitrap was operated with a positive ion spray voltage of 2.1 kV and a transfer tube temperature of 300C. The scan sequence began with an MS1 spectrum (Orbitrap analysis, resolution 120,000; 375–1575 m/z, AGC target 1 × 10^4^, maximum injection time 50 ms). The maximum number of precursors within a 3 s cycle time were fragmented using of collision-induced dissociation (normalised collision energy 35%) and the fragmented ions were analysed in the ion trap (turbo mode; maximum injection time 50 ms). To quantify the TMT reporter ion, we collected MS3 spectra using a method in which the top 10 MS2 ions were fragmented using HCD (collision energy 65%). The MS3 were analysed in the Orbitrap (AGC 1 × 10^5^; maximum injection time 105 ms; isolation window 2 m/z; resolution 50,000; scan range m/z 100–500). All acquisition methods are available in a summary format, as Additional file 1: Figure S2 (LFQ) and Additional file 1: Figure S3 (TMT MS3).

### Protein identification and quantitation

The raw spectral data were processed using MaxQuant [32] (version 1.6.5.0). For the LFQ data, the Andromeda search engine [33] integrated into the MaxQuant framework performed the spectral data search against a matched protein database (Additional file 2: Table S1) composed of 12 genome-derived proteomes for the bacterial species (downloaded 26^th^ November 2018 from NCBI) and 6 UniProtKB-derived protein databases for 5 fungal species and mouse (downloaded 30th December 2018). The MS3 spectra were searched against the mouse database.

For the MS data search, enzyme specificity was set to trypsin, allowing N-terminal cleavage to proline, and for ≤ 2 missed tryptic cleavages. Default settings were used for the MaxQuant searches, except that lysine acetylation and glutamate/glutamine conversion to pyroglutamic acid were set as variable modifications. N-terminal acetylation, methionine oxidation, and carbamidomethylation of cysteines was set as fixed modifications. The initial allowed mass deviation of the precursor ion was set to ≤ 20 ppm, and the allowed value for the fragment mass was set to ≤ 0.5 Da. Match between runs was used, with match time window 0.7 min and alignment window time 10 min. The maximum false discovery rates (FDR) at peptide and protein levels were set to 1%. Proteins LFQ intensities were determined by using the MaxLFQ algorithms [34], where the normalisation is applied on the whole dataset. This LFQ approach is based on accurate determination of spectrometric signal intensities (extracted ion chromatograms) of peptides and relies on measurements of the three-dimensional space of peptide ion intensity, *m*/*z*, and chromatographic elution time. However, for proteins at low abundance, XICs are often contaminated by nearby signals, and although a protein can still be identified, it might not be quantified because of low-quality data. TMT-MS3 data were processed with MaxQuant using Reporter ion MS3 and TMT6plex-Lys126-131 internal labels, with the search setting described above.

### Proteins filtering and functional analyses

The MaxQuant output data were analysed by using Perseus (version 1.5.6.0) [38]. Annotations for the identified proteins were downloaded from the UniProtKB database [35]. Protein functional analyses, including metabolic pathway analysis, were performed using the DAVID [36] and STRING-db [37] tools. Information on the significant differentially produced proteins derived from jejunal tissue was used to query the DAVID knowledge database for pathway enrichment analysis, using the mouse genome as a reference list.

Filtering of protein identifications was performed as follows: first, we applied a default filtering of MaxQuant search results on proteins marked as “reverse”, “only identified by site”, and “potential contaminant”. Next, only proteins identified in at least two biological replicates were considered. To identify false-positive identifications of microbial proteins in mice groups not colonised with bacteria and/or fungi, and previously ascertained by microbiological assays and quantitative PCR [29], we checked the protein identification type and filtered out those identified by the “match between runs” algorithm of MaxQuant and not directly identified by MS. Also, proteins identified only by a single peptide and not identified by unique peptides were removed. Any other remaining proteins, whose origins were not consistent with the type of microbial colonisation of a specific mice group were checked at the peptide level, to confirm whether these are valid identifications or not. For protein quantification, we considered only proteins with LFQ intensities in at least two biological replicates and identified by two unique peptides.

### Statistical analyses

We performed LC–MS/MS analyses from 3–4 replicates of pooled fecal samples collected from mice belonging to each treatment group that underwent the same microbial colonisation and was housed in the same cage and gnotobiotic isolator. Jejunal tissue samples were derived from 20 animals (5 GF, 5 B, 5 BY, and 5 Y mice), and the TMT experimental design is described in Additional file 1: Figure S1. To assess the biological variability of each experimental group, we calculated the Pearson correlation coefficients based on the protein intensities of each sample (Additional file 2: Table S2). LFQ intensities were derived by the MaxLFQ algorithm, onto which we further applied a median-centring normalization strategy. Before the normalisation step and statistical analyses, the proteins LFQ intensities were log_2_-transformed. To correct for differences in the sample amounts injected into LC–MS/MS, we normalised the relative protein amounts by dividing each protein LFQ intensity by the median intensity for all proteins in a given replicate [38] (Additional file 1: Figure S4). To account for potential peptide loading differences in fractions of the 4 different 6-plex TMT batches, we applied a correction factor based on the pooled samples containing TMT^6^ label 131 (Additional file 1: Figure S1). The TMT spectra intensities were first median normalised as described above and then each relative protein amount was divided by the correction factor.

To identify proteins with levels that differ substantially among the strains, we performed analysis of variance (ANOVA) to compare the global mean level of each protein against its corresponding amount in each condition. For the ANOVA test, FDR calculations were performed in Perseus by a permutation-based procedure with 250 randomisations and a cut-off of 5%. To determine the exact pairwise differences in protein levels, Tukey’s honestly significant difference (THSD) was performed on ANOVA-defined significant hits. Each species was analysed separately, and only relevant mice groups were used in the statistical analyses (e.g., only those colonised with bacteria were used to analyse bacterial proteins). Principal component analysis (PCA) was performed by using MetaboanalystR 3.0 [39], using log-transformed data normalised by median. Hierarchical clustering analysis based on the protein levels was performed in Perseus, by using Euclidean distance, average linkage, no constraints, and pre-processing with k-means. Figures 1 and 5 were created using diagrams from BioRender.com. Data are available via ProteomeXchange with identifier PXD019355.

## Results

### Fecal metaproteome of defined gnotobiotic model.

Using a shotgun LFQ approach, we profiled the fecal metaproteomes of germ-free mice (GF), and mice colonised with either 12 bacterial species (B—bacteria), five fungal species (Y—yeast), both bacteria and fungi (BY), and the latter group treated with an antibiotic (BY_ABX) or antifungal (BY_AFX) (Fig. 1A). We enriched microbial cells from feces of four-week-old mice by differential centrifugation, digested cell lysates by trypsin, and analysed the resulting peptide mixtures using LC–MS/MS. We queried the acquired mass spectra (5.3 × 10^6^ MS/MS) against a combined protein database of the 17 microbial species and mouse (Additional file 2: Table S1). The proteomic search identified 70,190 unique peptide sequences (Additional file 3: Table S3), mapped to 6675 proteins (Additional file 2: Table S4). Of these, 68% were bacterial, 22% fungal, and 10% were mouse proteins (Fig. 1B). Up to 58% of all detected proteins were characterised by LFQ intensities using the MaxLFQ method [34]. Principal component analysis based on the protein relative levels identified three main clusters (Fig. 1C), suggesting similar protein profiles of Y and GF, B and BY, and BY mice groups treated with antimicrobials (BY_ABX and BY_AFX).

From the bacterial proteomes, 4.4% of detected proteins shared peptides with other protein groups (Additional file 2: Table S5). However, most of the identified peptides belonged to a single protein group (leading protein group); that is, the sequence coverage of peptides unique for the leading protein group was considerably higher than the sequence coverage of peptides belonging also to alternative protein groups. For the fungal proteomes, a high percentage of identified peptides belonged to multiple homologous proteins from different strains of the same species (*e.g*., *Candida albicans* strains SC5314, WO-1, CD36) (Additional file 2: Table S5). This was due to the use of UniProtKB reference protein databases for specific fungal species (Additional file 2: Table S1), which included several strains of the same species. The use of reference databases was necessary because the fungal strains used for the gnotobiotic mice colonisation were clinical isolates without sequenced genomes, contrary to the bacterial strains, which have their genome sequence determined [20]. The shortcomings of using not perfectly matched protein databases were most apparent for two fungal strains (*Pichia kudriavzevii* and *C. krusei*), which were recently re-classified as strains of the same species [40] and did not have available individual databases. For these fungi, we used a common database based on reference *P. kudriavzevii* strains available in UniProtKB, resulting in decreased specificity of the MS data searches. Also, horizontal gene transfer might explain some of the homologous proteins; however, these events have been speculated to be relatively infrequent among microbial eukaryotes [41].

Using genome-specific protein databases for the bacterial stains also had the advantage of accurate assignment of proteins for the 12 different bacterial strains, for which we did not observe any species cross identifications. Nevertheless, the general UniProtKB databases did not compromise the accuracy of fungal and mouse protein identification, and we observed minimal cross-kingdom identifications (*e.g*., same peptides mapped to a fungal and mouse protein), with these proteins being manually filtered out.

### Inter-kingdom interactions influence the proteome response of gut bacterial species to antimicrobials

From 12 bacterial strains used for the gnotobiotic mice colonisation, *Clostridium clostridioforme* YL32 had the most detected proteins (Fig. 1B), while *Akkermansia muciniphila* YL44 had the highest predicted proteome coverage (33%) (Additional file 2: Table S5). The latter result correlated with 16S rRNA amplicon sequencing data [18], which determined *A. muciniphila* YL44 as the most abundant bacterial species. The number of proteins detected per condition was relatively similar for each species (Additional file 1: Figure S5).

From 2860 quantified bacterial proteins, we identified 1317 proteins whose levels significantly varied between the mice groups (ANOVA followed by THSD post-hoc analysis, FDR 5%, see Additional file 2: Table S6 for all proteins used for statistical analyses). For four bacterial species with the largest number of detected proteins (*C. clostridioforme* YL32, *A. muciniphila* YL44, *Blautia coccoides* YL58, *Muribaculum intestinale* YL27), about 50% of the quantified proteins showed differential levels (Additional file 2: Table S5), prompting in-depth evaluation of the strains’ protein profiles.

**Fig. 2:**
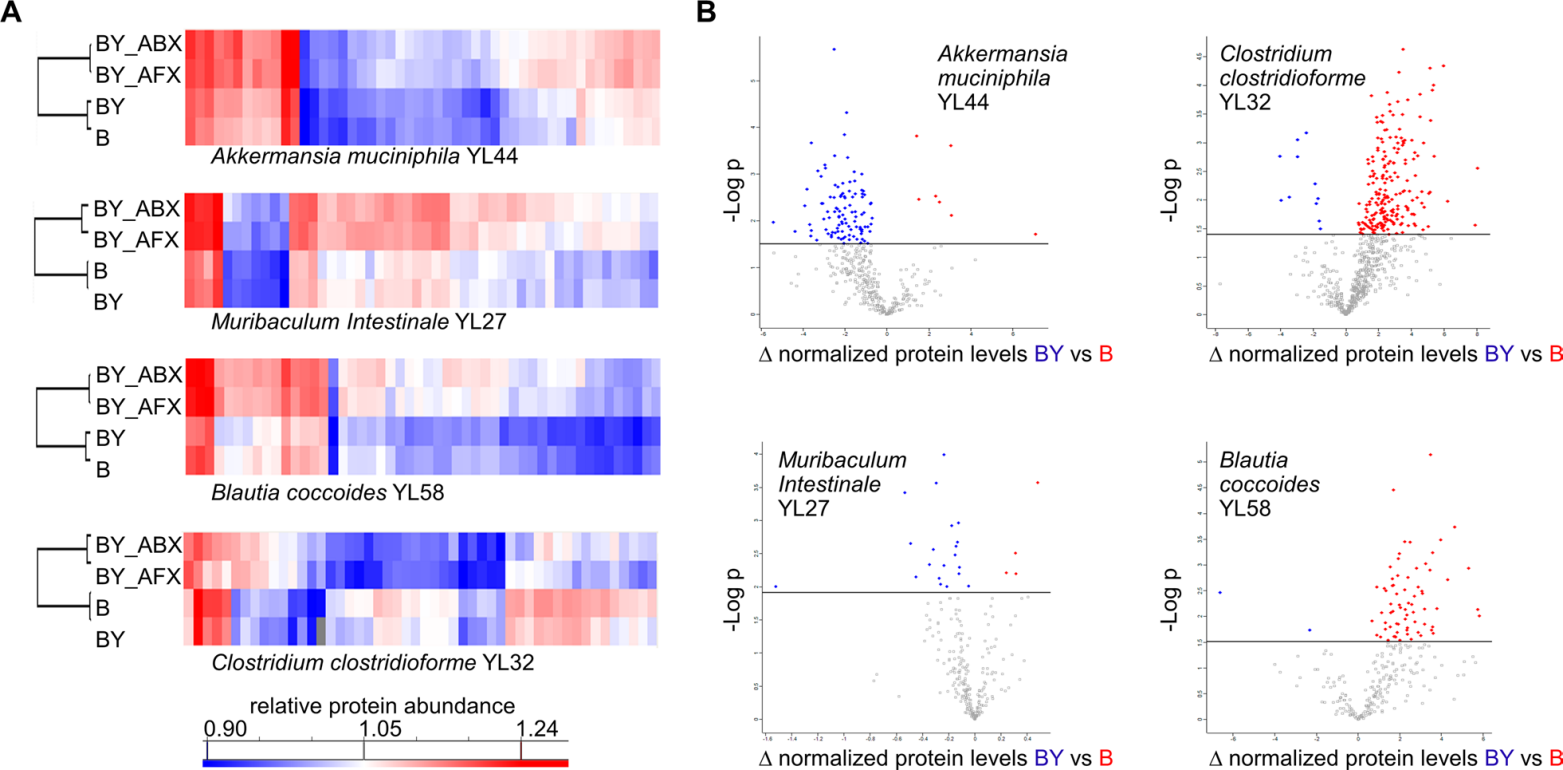
The response of four bacterial proteomes to treatment with antimicrobials and the presence of gut fungal species. **A** A selection of 50 most significant differentially produced proteins for *A. muciniphila* YL44, *M. intestinale* YL27, *B. coccoides* YL58, and *C. clostridioforme* YL32 (ANOVA, FDR 0.05). **B** Volcano plots representing the results of student t-test statistical comparison in protein levels between B and BY mice groups (FDR 0.05). Abbreviations of the mice treatment groups: B, bacteria; BY, bacteria-yeast; BY_ABX/AFX, bacteria-yeast and antibiotic or antifungal treatment

The proteomes of these bacterial species displayed an array of responses to antimicrobial-induced ecosystem perturbances. Figure 2A shows 50 of the most significantly changed proteins for four bacterial species with the largest number of detected proteins (see list of proteins and their functional annotation in Additional file 2: Table S7). In the presence of fungal species, antibiotic or antifungal treatment appeared to have a similar effect on the proteome of *A. muciniphila* YL44 (comparison of BY_ABX vs. BY_AFX groups), while for *M. intestinale* YL27, *B. coccoides* YL58, and *C. clostridioforme* YL32, the antibiotic treatment led to more proteins with increased levels compared to the antifungal groups (Additional file 1: Table S8). For *A. muciniphila* YL44, *M. intestinale* YL27, and *B. coccoides* YL58, many proteins had significantly increased levels in response to treatment with either antifungal or antibiotic (B/BY compared to ABX/AFX). For *C. clostridioforme* YL32, the detected proteome showed a contrasting response to the antimicrobial treatments. For instance, compared to the fecal proteome of the B only condition, there were 2.6 and 1.3 times less proteins with elevated levels than in the BY_AFX and BY_ABX groups, respectively (Additional file 1: Table S8). But when fungal species were present in the mouse gut (BY group), the effect of the antibiotic on *C. clostridioforme* YL32 appeared similar as for the other three strains (BY compared to BY_ABX), while treatment with antifungal led to a 1.3-fold increase in proteins with elevated levels in the BY group (BY compared to BY_AFX).

It should be noted that the effect of increased protein levels in the ABX/AFX groups could be further amplified by the MS detection method itself, which is less sensitive to low abundant proteins and will preferentially identify those with higher abundances (see Additional file 2: Table S1—Summary of MS data search). In any case, these comparisons indicate that antimicrobial perturbance targeting bacterial or fungi cause differential responses among bacterial species that depend on species identity and presence or absence of fungi.

For the rest of the bacterial strains, low numbers of quantified proteins did not allow for in-depth investigations. Nonetheless, seven of the strains showed considerable differences in the protein profiles between the mice groups (Additional file 1: Figure S6). A general picture has emerged from this comparison, in which antimicrobial treatments targeting bacteria or fungi during the second week of the mice’s life resulted in lasting changes in the proteomes of the gut bacterial species.

### Gut fungi differentially modulate bacterial proteomes

Next, we statistically compared the bacterial protein levels between the B and BY groups to investigate the effect of fungi on the proteomes of the four strains with the highest numbers of detected proteins (Fig. 2B). Similarly to what we observed for antimicrobials, fungal presence diversely influenced the proteomes of individual bacterial species. *A. muciniphila* YL44 and *M. intestinale* YL27 displayed increased amounts of proteins from various functional classes in the presence of fungi (Additional file 1: Figure S7). We observed the opposite for *C. clostridioforme* YL32 and *B. coccoides* YL58, where the levels of 100 out of 107 and 51 out of 52 differentially produced proteins, respectively (t-test, FDR 5%), decreased when fungi were present (Additional file 1: Table S8). The proteomic observations for *A. muciniphila* YL44 appeared to be inversely correlated with a decrease in *A. muciniphila* abundance based on 16S rRNA sequencing in the presence of fungi [18] (Additional file 1: Figure S7). For *C. clostridioforme* YL32, the trend of increased protein levels in the B group compared to the BY group also appeared to inversely correlate with the 16S-based abundances; however, these trends did not reach statistical significance in the 16S sequencing data. Similarly, we could not derive a correlation with the sequencing data for *M. intestinale* YL27 and *B. coccoides* YL58, as the strains’ relative abundance appeared similar across the mice groups. Metaproteomic analyses thus provided complementary information to the sequencing data and yielded a higher resolution of the ecological interactions between the gut microbial species.

### Gut bacteria and antimicrobial treatments modulate fungal proteomes

**Fig. 3:**
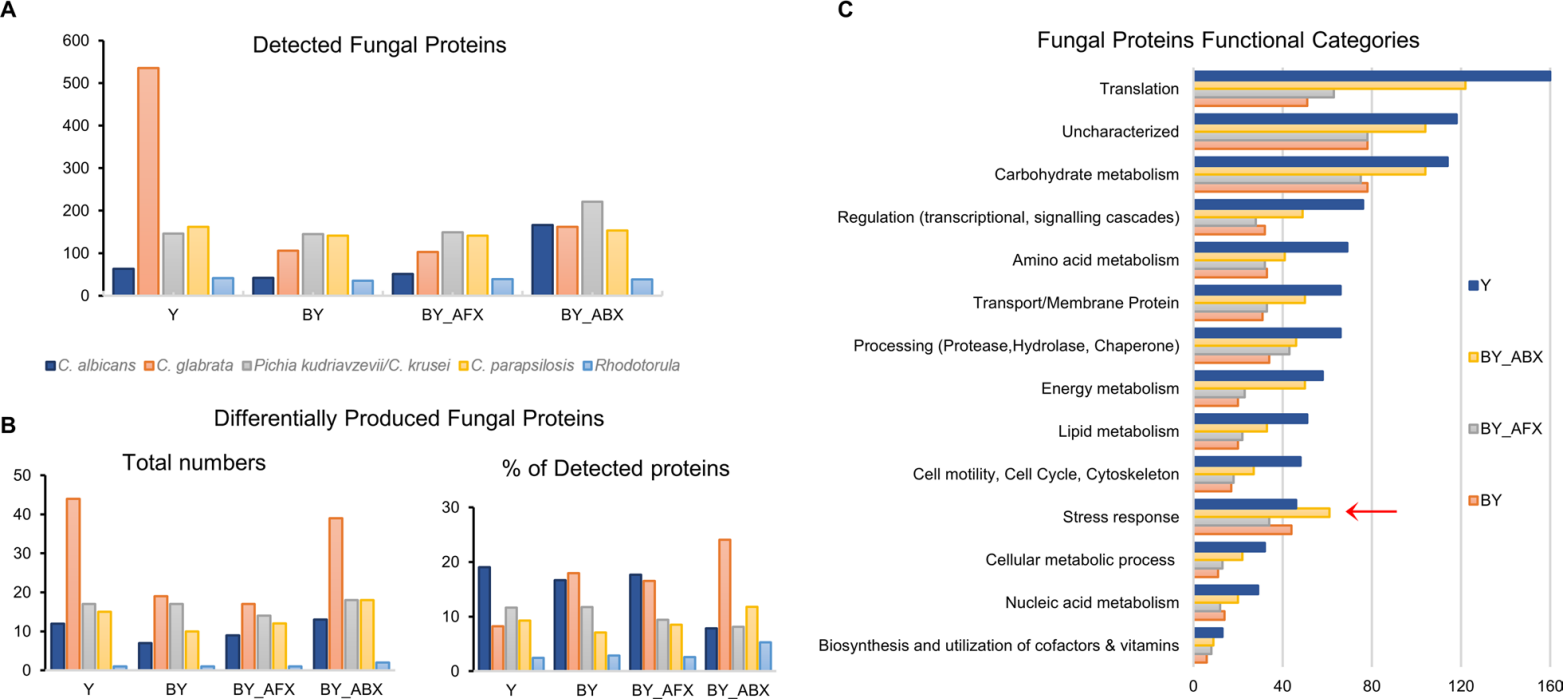
Response of fungal proteomes to antimicrobials and presence of bacteria. **A** Distribution of detected fungal proteins across four mice group (n = 1492). **B** Distribution of fungal proteins with significantly changed levels across four mice groups (n = 99, ANOVA, FDR 5%). Additional file 2: Table S6 shows the fungal proteins identified as having significantly different levels. **C** Distribution of the main functional classes of detected fungal proteins (n = 1492). Proteins functional annotation was downloaded from the UniProtKB database [29] and compared to annotations obtained using the DAVID [30] and STRING-db [31] tools. Abbreviations of the mice treatment groups: BY, bacteria-yeast; BY_ABX/AFX, bacteria-yeast and antibiotic or antifungal treatment; Y, fungi/yeast

We detected 1492 proteins for the five fungal species colonising the gnotobiotic mice, and 39% of these proteins were assigned LFQ levels. The lower numbers of proteins detected and quantified, as compared to bacteria, were a result of a lower abundance of fungi in the gut that we previously confirmed by quantitative PCR and microbiological assays [18]. Also, the LFQ approached used [34], is less sensitive to low abundant proteins, which are less likely to be quantified. Most of the identified fungal proteins were present in the Y group representing the mice colonised exclusively with fungi. We previously showed that the Y group harboured higher fungal concentration than co-colonised mice [18], confirming that a bacterial suppression of the fungal colonisation occurs in the host gut [42]. Of the five fungal species, *C. glabrata* had the highest number of identified proteins, which also dominated the Y group (Fig. 3A). However, antibiotic treatment (BY_ABX) led to higher detection of proteins derived from *Pichia kudriavzevii*/*C. krusei*. The total number of differentially abundant proteins between the mice groups and their distribution among the fungal species were rather similar for the following pairs of mice groups: Y—BY_ABX and BY—BY_AFX (Fig. 3B). When we expressed the number of differentially produced fungal proteins as a percentage of each strains detected proteins, we noticed that *C. glabrata* proteome showed a stronger response to the antibiotic treatment (BY_ABX), as compared to Y, BY and BY_AFX.

The presence of bacteria and early-life antimicrobials had significant effects on the fungal species protein profiles. Arrangement into categories using functional annotations based on UniProtKB [35] and other databases [36, 37], showed an increased number of fungal proteins related to stress responses in the antibiotic condition (Fig. 3C), suggesting that the fungal species are either negatively affected by the changes in the bacterial microbiome or by the antibiotic treatment itself. Interestingly, many of the identified fungal proteins were previously reported to be excreted via extracellular vesicles [43, 44] and to have immunogenic properties [45, 46] (Additional file 2: Table S9). Among these proteins were enzymes of the glycolytic pathway (*e.g*., enolase, glyceraldehyde-3-phosphate dehydrogenase, fructose-bisphosphate aldolase, and phosphoglycerate mutase) and molecular chaperones linked to stress response (heat shock proteins). These proteins were significantly elevated in the fungal group suggesting that they are critical cytosolic proteins abundantly produced by fungal cells living in the mouse gut.

### Fungal and bacterial colonisation induces distinct and persistent changes in the host fecal proteome

**Fig. 4:**
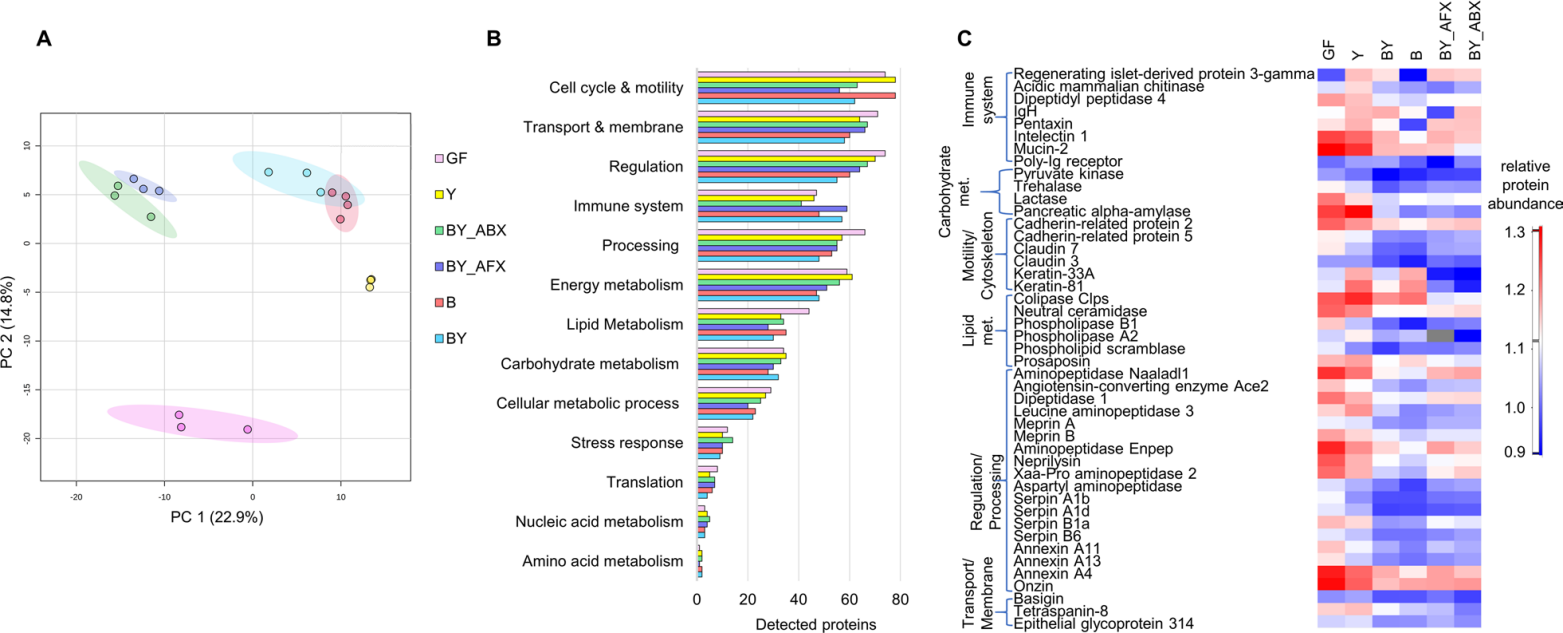
The response of host fecal proteome to microbial colonisation and antimicrobial treatment. **A** PCA score plot based on 405 quantified mouse proteins. X- and Y-axis show the first and second principal components, accounting for 22.9% and 14.8% of the total variation, respectively. **B** Functional classes of 651 detected mouse proteins, with the protein annotations derived from the UniProtKB database [29] and compared to those obtained by the DAVID [30] and STRING-db [31] tools. **C** Selection of differentially produced mouse proteins extracted from feces. Abbreviations of the mice treatment groups: B, bacteria; BY, bacteria + fungi; BY_ABX/AFX, bacteria + fungi and antibiotic or antifungal treatment; GF, germ-free; Y, fungi/yeast

We identified 674 mouse proteins as part of the mice fecal metaproteome. From proteins quantified by LFQ (405), 71% displayed differential abundance between the groups (ANOVA followed by THSD post-hoc analysis, FDR 5%, Additional file 2: Table S10). PCA score plot based on the quantified proteins showed four clusters (Fig. 4A): the first contained co-colonised mouse groups treated with antimicrobials (BY_ABX/AFX), and the second consisted of the B and BY groups. The third and fourth clusters included the Y and GF groups, and these were considerably more dissimilar from the first two clusters. Host proteins in the Y group were more clearly separated from the GF mice, in contrast to clustering when also microbial proteins were considered (Fig. 1C), revealing that the proteome response to fungal colonisation may be more pronounced in the host proteome than in microbial cells. The GF group displayed the highest number of proteins associated with lipid metabolism, regulation, and molecular processing, while the BY and BY_AFX groups had the highest numbers of immune proteins (Fig. 4B). In the BY_ABX group, we identified an increased number of stress response proteins. Moreover, the Y and GF groups displayed an increased number of proteins associated with energy metabolism (*e.g*., mitochondrial proteins).

Proteins whose levels significantly varied between the mouse groups belong to the following functional classes: processing proteins such as proteases, hydrolases, and chaperones (14%), proteins linked to cell cycle regulation, motility, and cytoskeleton, including components of the tight junctions (14%), transport and membrane proteins including epithelial cell surface receptors (13%), proteins of energy and lipid metabolism (12% and 9%, respectively), and gut immune and barrier factors (9%). To tease apart the mouse proteome response to different microbes, we statistically compared pairs of treatment groups containing either fungi or bacteria (Additional file 1: Figure S8). The introduction of the 12-species bacterial consortium led to a striking decrease in the mouse protein levels, and this was valid both for germ-free mice (GF vs. B) and mice colonised with fungi (Y vs. BY). Although gut fungi did not have the same significant effect on the mouse proteome as bacteria, we detected a fivefold decrease in the number of proteins whose levels increased in the Y group compared to GF (GF vs. Y). The presence of fungi and bacteria resulted in a twofold increase compared to bacteria alone (BY vs. B), particularly of proteins associated with the immune system, energy metabolism, and cellular processing.

Because the mouse protein levels were strongly dependent on the gut microbial consortium, we gave special attention to the individual functional classes of differentially produced proteins. Figure 4C shows 45 differentially produced mouse proteins with the largest variation between the mice groups. Levels of 27 immune proteins were the highest in GF mice, followed by those colonised only with fungi. Two immune proteins were elevated in the presence of fungi: acid mammalian chitinase (Chia), implicated in the defense response against fungi, and regenerating islet-derived protein 3-gamma (Reg3g), a bactericidal C-type lectin reported to have bactericidal activity [47, 48]. Thus, our results suggest that Reg3g might also be involved in the intestinal cells’ response to fungal colonisation. However, more data will be needed to support the involvement of Reg3g in the epithelial cell response to fungi.

Bacterial presence resulted in a reduction in the mucosal Pentaxin (Mtx2) levels, a secreted protein involved in complement activation, Intelectin 1 (Itln1), a receptor that binds microbial glycans, and Dipeptidyl peptidase 4 (Dpp4), cell surface receptor and dipeptidyl protease involved in T-cell activation. In addition, levels of other immune proteins, such as Mucin 2 (Muc2), Poly-Ig receptor (Pigr), and immunoglobulin heavy chain (IgH), were altered by the shifts in microbial composition caused by antimicrobial treatments.

The absence of microbes resulted in elevated levels of mouse proteins linked to carbohydrate metabolism both at the cellular [e.g., Pyruvate Kinase (Pkr)] and host level [glycosidases such as Pancreatic Alpha-Amylase (Amy2), Lactase (Lct), and Trehalase (Treh)]. Amy2, a glycosidase that hydrolyses alpha-linked polysaccharides such as starch and glycogen, had the highest levels in the Y group, suggestive of increased Amy2 production response to either fungal polysaccharides or fungal metabolism on dietary sugars. Fungi also elicited a strong effect on host proteins associated with lipid metabolism. The highest levels were identified in the Y group for the host enzyme Colipase (Clps), a cofactor of pancreatic lipase facilitating lipids digestion, and Phospholipase A2 (Pla2g1b), a secreted protein involved in lipid degradation and innate immune mucosal response. Closely connected to the immune system are phospho- and sphingolipid metabolism enzymes, which often have regulatory properties towards immune cells. Of those, we identified Neutral Ceramidase (Asah2), a membrane protein hydrolysing sphingolipid ceramides into sphingosines and free fatty acids, whose relative levels were the lowest in BY and B groups.

Cytoskeletal and cell adhesion proteins were also significantly increased in the GF and Y mice. These included cadherin-related proteins 2 and 5 (Cdhr2 and Cdhr5), which regulate microvilli length, and Claudins 3 and 7 (Cldn3 and Cldn7), controlling tight junction-specific obliteration of the intercellular space. Notably, Keratin-33A and -81 (Krt33a and Krt81), structural proteins that form the cytoskeleton’s intermediate filaments in epithelial cells, displayed the highest levels in the Y group, followed by the B group and had the lowest levels in the BY groups treated with antimicrobials. Adhesion proteins with regulatory properties and links to the immune system included Tetraspanin-8 (Tspan 8), Epithelial Glycoprotein 314 (Epcam), and Basigin (Bsg), a cell surface receptor. All three proteins showed increased levels in the GF and Y groups.

One of the most prevalent functional classes among the differentially produced proteins were proteases, which had the highest levels in the GF and Y groups, and the lowest in the BY and B groups. Among these was for example Angiotensin-converting enzyme (Ace2), a carboxypeptidase with multiple regulatory functions, including gap junction assembly. Other proteases with regulatory properties included membrane-bound metalloproteases Meprin A (Mep1a) and B (Mep1b), implicated in the inflammatory response. Alongside proteases, protease inhibitors followed the same quantitative pattern as described above. These included Serpins, serine protease inhibitors that negatively regulate endopeptidase activity in response to cytokines (Serpinb1a, Serpina1d), innate immune response, inflammation, and cellular homeostasis (Serpinb1a, Serpinb6).

Numerous regulatory proteins were present among the proteins with increased levels in the GF and Y group, such as the Annexin family of Ca^2+^-regulated phospholipid-binding and membrane-binding proteins (Anxa4, Anxa11, Anxa13) and nuclear proteins such as Onzin (Plac8) suggested to regulate immune responses. Overall, the metaproteomic analyses documented an extensive impact of microbial colonisation in a controlled early life gut microbiome model, underlining the intertwined functional development of the host and its gut microbiome, and revealing new features of the host response to fungal colonisation.

### Fungal colonisation drives alterations in the mouse jejunal tissue proteome

**Fig. 5:**
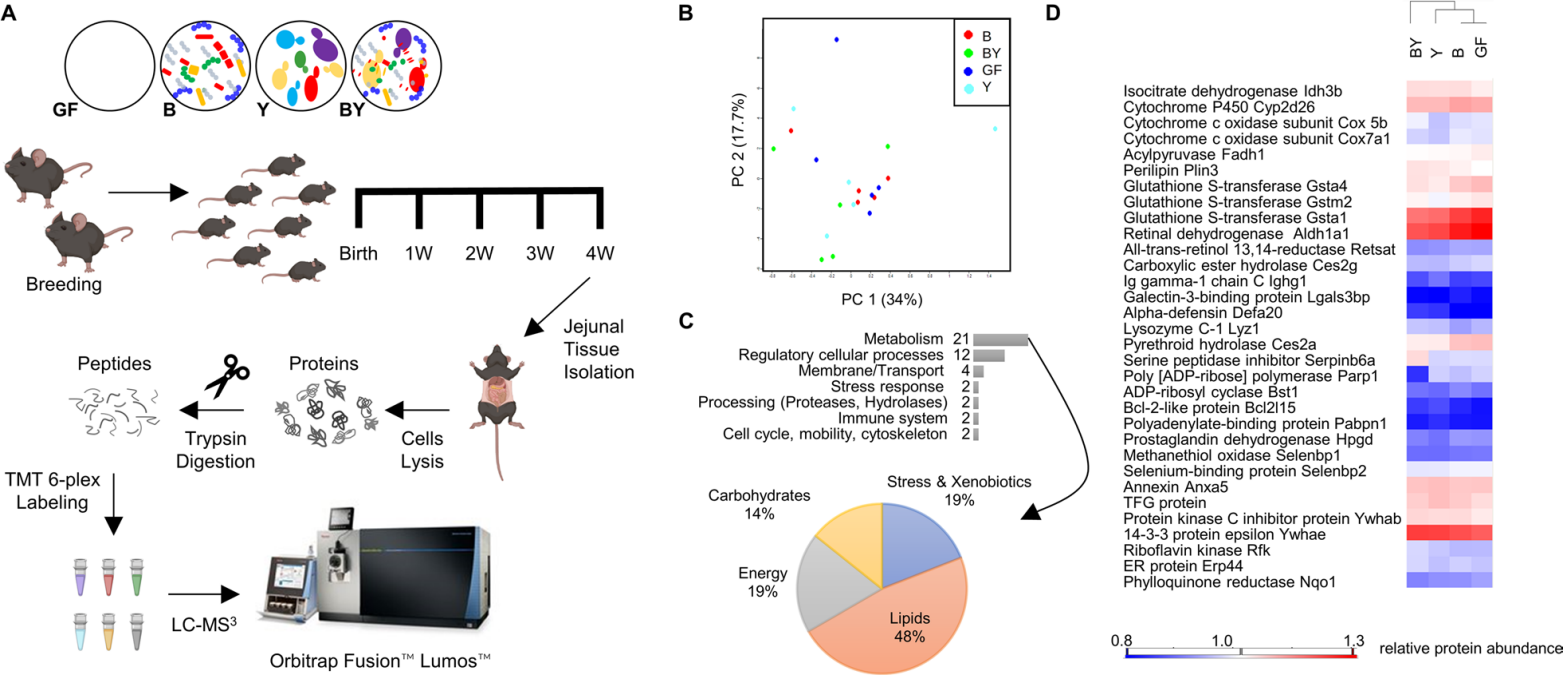
Response of mouse jejunal proteomes to microbial colonisation. **A** Experimental design of Tandem Mass Tag (TMT) labelling experiment. **B** PCA score plot based on 1019 mouse proteins quantified in a minimum of 5 biological replicates. X- and Y-axis show the first and second principal components, accounting for 34% and 17.7% of the total variation, respectively. **C** Functional classes of 45 differentially produced mouse jejunal proteins (ANOVA followed by THSD, FDR 5%). **D** Selection of differentially produced mouse proteins extracted from jejunal tissue. Abbreviations of the mice treatment groups: B, bacteria; BY, bacteria + fungi; GF, germ-free; Y, fungi/yeast

Given the exciting findings from the mice fecal metaproteomes, we decided to investigate more closely the host proteome. We selected the small intestine as our site of interest because it contains the body’s largest immune organ (the gut-associated lymphoid tissue) and is an important site of the host-microbe interactions [49]. We used a tandem mass tag labelling approach (TMT 6-plex) to gain a higher sensitivity for low abundant proteins (Fig. 5A). From the TMT 6-plex experiment, we identified 10,201 peptides (Additional file 2: Table S11) matched to 1514 mouse proteins (Additional file 2: Table S12), and the levels of 45 were significantly altered between the mice groups (ANOVA, THSD post-hoc analysis, FDR 5%, Additional file 2: Table S13). PCA score plot revealed different grouping results than for the mouse fecal proteomes (Fig. 5B). Functionally, the 45 significantly changed proteins were mainly associated with cellular metabolism and regulation of cellular processes (Fig. 5C). Pathway analysis identified oxidation–reduction, glutathione metabolism and cell–cell adherence as significantly enriched processes (Additional file 2: Table S14).

Compared to the fecal mouse proteome, proteome changes of the jejunal tissue related to microbial colonisation were more subtle. Figure 5D shows the relative levels of 45 differentially produced mouse jejunal proteins with significant variation between the mice groups. Among immune proteins whose levels increased with the presence of fungi were Alpha-defensin 2 (Def20) and Ig gamma-1 chain C region secreted form (Ighg1). From 10 proteins functionally connected to lipid metabolism, two Glutathione S-transferase enzymes (Gstm2 and Gsta4) were significantly decreased in the fungal group. We also detected two proteins of retinol metabolism (Retsat and Aldh1a1), which play a key role in mucosal immune responses. Fungal colonisation further appeared to impact the intestinal cells’ energy metabolism, as several mitochondrial proteins had decreased levels in the fungi group, most notably, two subunits of Cytochrome C oxidase (Cox5b and Cox7a1).

Some of the differentially produced proteins have functional links to NF-κB pathway, such as the apoptotic marker Poly (ADP-ribose) polymerase (Parp1). In some contexts, particularly in response to cellular stress, stimulation of the NF-κB pathway promotes apoptosis [50]. Selenium acts as a key element that controls NF-κB activation and the half-life of its inhibitor IκBα, and we detected two selenium-binding proteins (Selenbp1 and Selenbp2). Overall, the TMT 6-plex analysis of the jejunal tissue provided further insight on host cellular pathways impacted by defined microbial colonisation and confirmed that gut fungi elicit differential effects compared to bacteria.

## Discussion

In this work, we used a controlled environment of gnotobiotic mice colonised with defined microbial consortia to precisely describe changes in the metaproteome profiles associated with alterations in the gut microbiome. By reducing the gut microbiome’s complexity to 12-bacterial species, five fungal species, or the combination of the 17 microbes, the gnotobiotic model allowed us to isolate specific contributions and cross-kingdom interactions between the gut-associated bacteria and fungi, as well as the host response to the microbial colonisation.

Several normalisation methods for quantitative metaproteomic data have been suggested, including generation of a mock community, and normalisation of protein levels for each sample and species to a constant value [51]. The latter method depends on a sufficient number of measurements for each species to be normalised. We described in our earlier study [29], using DNA sequencing data, that some of the bacterial and fungal species are in low abundance. For that reason, we chose not to normalise the data for each species to a constant value because only very few proteins would be considered for the low abundant species and possibly skew the data in favour of the more abundant species. However, it is possible to avoid normalisation to species if the reference protein sequences of the organisms under study are used for generating quantitative proteomic data [51]. Indeed, we took advantage of the genome-sequenced bacterial consortium [20] and used matched protein databases for MS data searches. This strategy yielded an accurate and specific identification of proteins from the 12 bacterial species. For fungal strains that did not have sequenced genomes, the use of UniProtKB reference protein databases resulted in decreased specificity of the MS data searches, yet still correct assignment of fungal proteins to specific species. In this way, we avoided the issue of protein identifications shared between two or more species, which would compromise the accuracy of the determination of the protein’s relative quantitative levels and prevent its use in subsequent statistical analysis. Moreover, the used MaxLFQ normalisation method [34] have been applied in other metaproteomic studies for accurate label-free quantification of proteins [52].

The fungal species chosen for the gnotobiotic experiment are amongst the ones commonly reported from human samples [1, 27, 53]. Still, we acknowledge that a limited number of species was investigated, and the effect of other important fungi (*Malassezia* sp., *Saccharomyces* sp.) remains to be tested. Our previous work determined the impact of the selected gut fungal species on the structure of gut microbiome community and host immune development [18]. Here, we strengthened these results using a metaproteomic approach that provided an independent functional measure of the microbiome-host crosstalk. Similarly to other multi-omics studies [54, 55], we found discrepancies between the quantitative proteome profiles and the DNA-based relative abundance of individual species. These differences likely stem from complex regulatory networks along the gene to protein expression path, which differ between microbial kingdoms. The latter was recently documented in a study showing dynamic ratios between protein and RNA levels that depended on the type of microbial population [56].

For the fungal species, a meaningful comparison between the protein and DNA data was hindered by the absence of genome-specific protein databases that would guide the raw MS data searches. However, corresponding with our earlier results [18], the proteomic data showed that fungi grew in higher concentrations in bacteria’s absence. This finding is in line with the interkingdom competition and antagonism between bacteria and fungi in other ecosystems, such as the rhizosphere and soil [57], and in mammalian hosts, where commensal bacteria limit fungal colonisation via activation of innate mucosal immunity [42] and directly by producing inhibitory metabolites such as short-chain fatty acids [58].

**Fig. 6:**
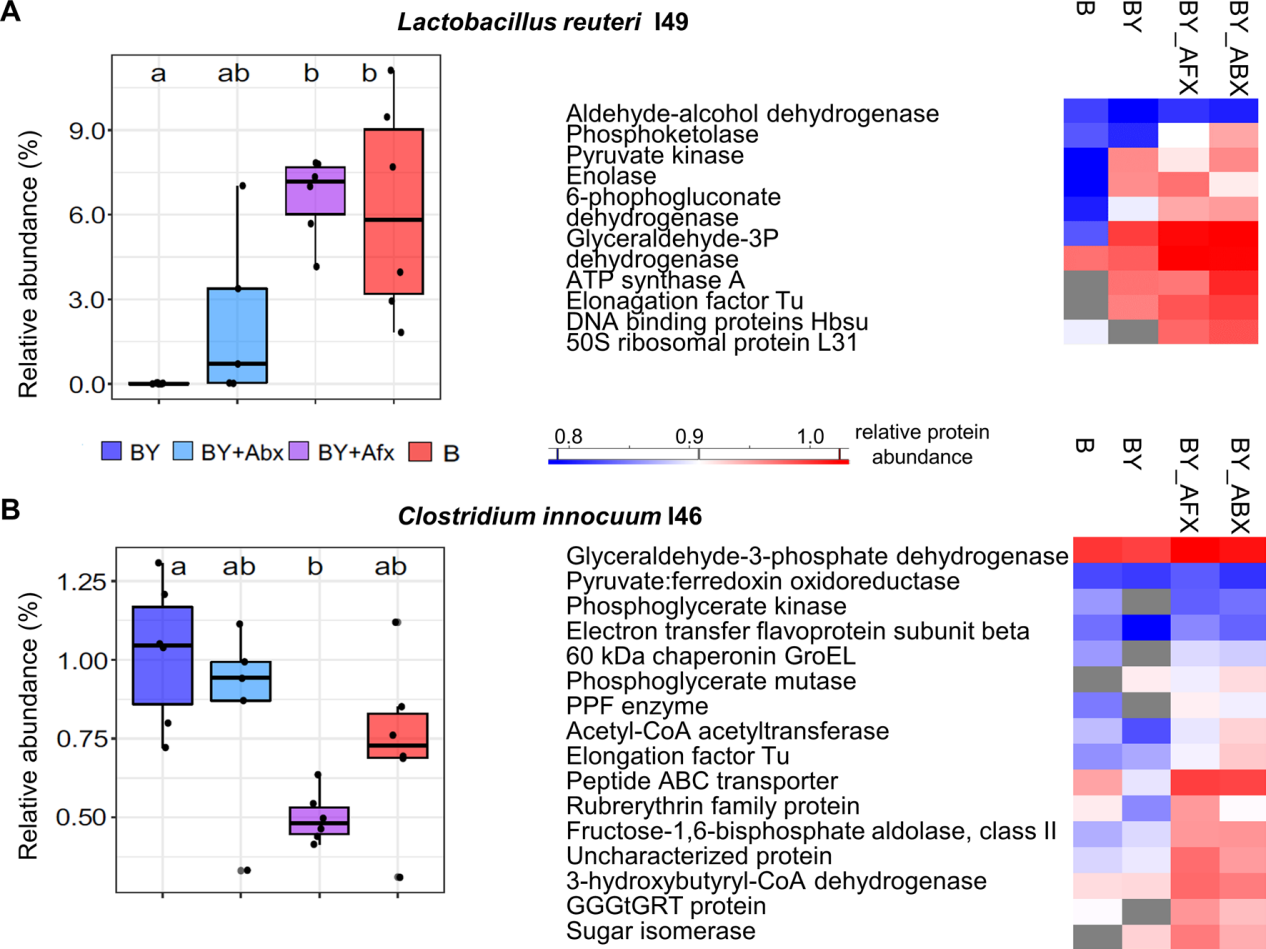
Detection of stress response in bacterial proteomes. The relative abundance based on 16S rRNA sequencing and differentially produce proteins of *Lactobacillus reuteri* (**A**) and *Clostridium innocuum* (**B**). Proteins quantified in a minimum of two replicates are shown; grey fields in heatmaps indicate the protein amount was below the quantification limit. Colour denotes microbial colonization (red B – bacteria, blue BY – bacteria + yeast, cyan blue BY_Abx – BY + antibiotic, purple BY_Afx – BY + antifungal)

Despite the lack of a linear relationship between the DNA and protein data, our earlier results from the 16S rRNA sequencing aided the interpretation of the bacterial proteome responses, and the combined analyses revealed antagonistic and synergistic relationships between the bacterial and fungal species. For example, we identified *Lactobacillus reuteri* as a potent responder to the fungal presence from the sequencing data, as the bacterium abundance was significantly reduced in the BY group. Only a few *L. reuteri* proteins were quantified, and those displayed lower amounts in the absence of fungi, conversely to the DNA sequencing results (Fig. 6A). Some of the most abundant bacterial proteins were detected in the B-only group (ribosomal protein L31, enolase, and translational factor Tu) along with three dehydrogenases, whose increased production often indicates cellular stress. For another low abundant bacterial species, *Clostridium innocuum*, we observed a similar reciprocal relation between the proteomic and sequencing data (Fig. 6B). Taken together with the observed variations in the proteome response of the four most abundant bacterial species (Fig. 2), the proteomic data is suggestive that the cells are affected by changes in the gut environment related to the fungal presence (*L. reuteri*, *A. muciniphila*, *M. intestinale*) or absence (*C. innocuum*, *B. coccoides*, *C. clostridioforme*). Similar results were found in samples from mice treated with antimicrobials, which impacted levels of numerous proteins and often led to their elevated production (Fig. 2 and Additional file 1: Fig. S6). Such proteome response has been documented for bacterial cells treated with antibiotics [59], where the cells optimise their proteins’ production to deal with the adverse environmental factor. Moreover, protein aggregation is a likely bacterial strategy to survive antibiotic treatment [60].

One of this study’s most striking findings was a similar impact of the antibacterial and antifungal treatments on bacterial and fungal species’ proteomes. Inhibitory effects of antifungal drugs on the growth of commensal bacteria are poorly described [61], but it may be that the antifungal Fluconazole inhibits intestinal bacteria to some extent via direct or indirect mechanisms, similarly to other non-antibiotic drugs [62]. The antibiotic and antifungal treatments also had a strong effect on the quantities and types of fungal proteins being produced, revealing how these antimicrobial drugs impact the entire community, with similar functional consequences. Ecosystem perturbances caused by either antibiotics or antifungal drugs may impact the gut microbiome keystone species, causing a widespread effect on microbial networks and food webs [63].

Host proteins present in the intestine are essential for maintaining a mutualistic relationship with the microbes on the mucosal interface and serve as reporters on the host-microbe interactions. Host fecal proteome has been reported to exhibit signatures specific to colonisation states [64]. However, to our knowledge, protein response to exclusive fungal colonisation has not been characterised before. For the first time, we described at the proteome level the cellular pathways and their protein components involved in the interactions between the mammalian host and gut fungal species. Fungal colonisation resulted in changes in host proteins functional in innate immunity as well as metabolism (Fig. 4), suggesting specific roles of gut fungi on host systems during early developmental stages. Further research aimed at investigating these roles has great potential for novel discovery, given that most host-microbiome interactions described to date have been limited to bacteria.

In the host jejunal proteome, most of the differentially produced proteins had metabolic functions (Fig. 5). The quantitative profiles of proteins with significantly changed levels showed closer clustering of the GF and B groups (Fig. 5D). These results likely reflect the host control of the number of bacterial cells in the small intestine, where they would otherwise compete for easily digestible nutrients. Alternatively, the more subtle differences in jejunal proteomes across microbial colonisation conditions, as compared to profound changes identified in fecal metaproteomes, may also reflect host homeostatic pressures present in intestinal tissues. Nonetheless, these findings indicate that fungal colonisation influences host jejunal proteome in a distinct way from bacteria.

The identification of multiple fungal proteins hypothesised to be abundant components of the extracellular vesicles suggests that fungi influence the host through direct cell-to-cell contact. Possible mechanisms may be akin to recently described interactions between segmented filamentous bacteria and mice intestinal epithelial cells [65]. These bacteria protruded into the epithelial cells and used adhesion-triggered endocytosis to transfer antigens into intestinal epithelial cells and modulate host T cell homeostasis. Similarly, epithelial internalisation of fungal hyphae, a morphology into which fungal cells transition to strengthen their adherence to epithelial cells [66], can deploy fungal extracellular vesicles with host cell-modulatory properties. Fungal cells produce a diverse array of biologically active compounds [67, 68], but the understanding of the mycobiome contribution to immunomodulatory substances present in the gut remains rudimentary.

It should be noted that the results of this experimental study need to be interpreted with caution, since only a limited number of microbial species was included. We used a selection of prevalent fungal species of the mammalian gut, yet many more remain to be explored and characterized. In addition, the complexity of interactions between the host and the gut microbiome, as well as within the different microbial kingdoms, will likely increase exponentially with an increasing number of species. Other limitations of this work include the lack of genome references for the fungal strains colonising the mice. Further efforts to carry out whole genome sequencing of fungal species and strains commonly associated with mammalian gut will reveal a better-resolved analysis of the functional role of these understudied microbes to their microbial ecosystems and their impact on the host. Also, fecal samples are a proxy for the colon microbiome. Although technically challenging, sampling along the gastrointestinal tract will reveal additional insights on microbial colonisation of the small and large intestine. Finally, label-free quantification is a common strategy adopted in metaproteomic studies but has low sensitivity to more scarce proteins as well as limitations in quantification accuracy. The application of metabolic labelling for improved peptide quantification [69] holds the potential to improve the accuracy of quantitative metaproteomics, and promote the application of proteomics for functional studies of intestinal microbiomes.

## Conclusions

In this work, we report previously unknown interactions between specific gut bacteria, fungi, and a mammalian host by using quantitative proteomic analyses of gnotobiotic mice colonised with defined microbial communities. Broad changes in microbial proteomes reflected interkingdom interactions between bacteria and fungi, as well as a response to antimicrobials. Our data also described for the first time the key role of fungal colonisation and how it impacts the host intestinal proteome. Further, we characterised cellular pathways and their protein components involved in the interactions between the mammalian host and gut fungal species. Our results suggest that an increased abundance of certain fungal species in early life may impact the developing intracellular balance of epithelial and immune cells. The work sets the stage for future studies that will explore the details of molecular mechanisms by which gut fungi modulate host physiology.

## Supplementary Information

**Additional file 1:** Supplementary information, including Figures S1–S8, and Table S8.

**Additional file 2:** Supplementary Tables S1–2, S4–S8, S9–S14.

**Additional file 3:** Supplementary Tables S3.

## Data Availability

Data are available via ProteomeXchange with identifier PXD019355.
