# Supplementary material for: Metaproteomic profiling of fungal gut colonization in gnotobiotic mice
Source: Anim Microbiome. 2022 Feb 22;4:14. doi: 10.1186/s42523-022-00163-2 (PMC8862486; doi:10.1186/s42523-022-00163-2)
Supplement: Supplementary file 1 — Additional file 1: Supplementary information, including Figures S1–S8, and Table S8. [file 42523_2022_163_MOESM1_ESM.docx]

**SUPPLEMENTARY INFORMATION FOR**

**Metaproteomic Profiling of Fungal Gut Colonization in Gnotobiotic Mice**

Veronika Kuchařová Pettersen ^a, b, c, d^, Antoine Dufour ^a^, Marie-Claire Arrieta ^a, c, d^

^a^ Department of Physiology & Pharmacology, University of Calgary, Calgary, Canada

^b^ Department of Pediatrics, University of Calgary, Calgary, Canada.

^c^ International Microbiome Centre, Cumming School of Medicine, University of Calgary, Calgary, Canada.

^d^ Department of Medical Biology, UiT The Arctic University of Norway, Tromsø, Norway

**TABLE OF CONTENT:**

**Figure S1** TMT labels carried by each sample and the mixing design

**Figure S2** Summary of the TMT MS3 acquisition method

**Figure S3** Summary of the LFQ MS/MS acquisition method

**Figure S4** Median normalisation of LFQ data

**Figure S5** Detected bacterial proteins per strain per condition

**Figure S6** Response of bacterial proteomes to antimicrobials and the presence of fungi (seven strains with a low number of quantified proteins

**Figure S7** Number and functional classes of differentially detected bacterial proteins in either B or BY mice groups for four bacterial species.

**Figure S8** Response of mouse fecal proteome to the presence of microbial consortiums.

**Table S8** Number of proteins with significantly increased levels for selected bacterial strains and between different mice groups


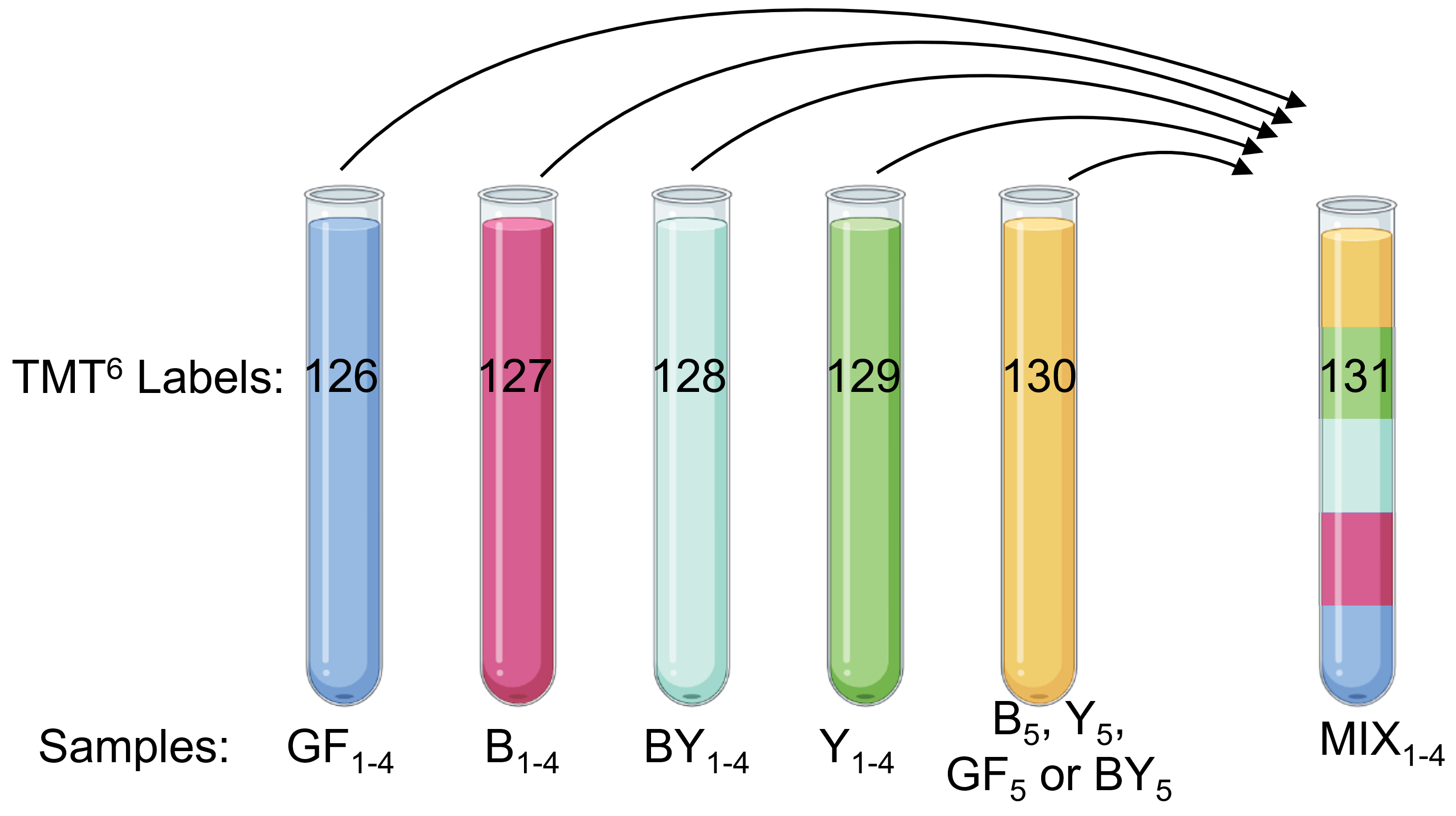


**Figure S1** TMT labels carried by each sample and the mixing design. Four TMTsixplex™ Isobaric Mass Tagging Kit were used for labelling of 20 samples and 4 pooled controls. Samples originated from jejunum of mice that were either left germ-free (GF), colonised with bacterial (B), fungi (F) or both (BY). A unique reporter mass (126-131 Da) of the TMT6 isobaric labels is described on each tube.


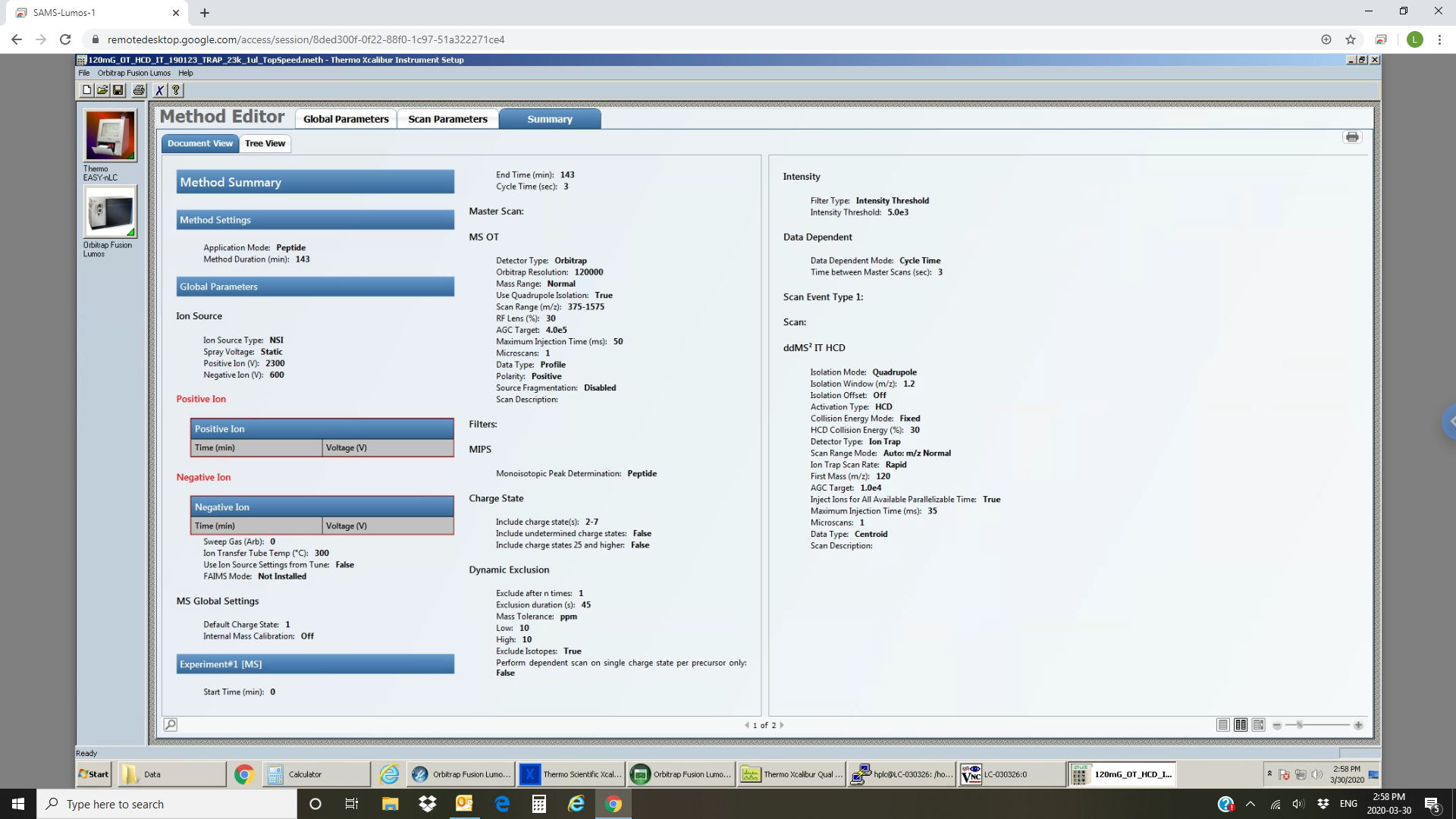


**Figure S2** Summary of the LFQ MS/MS acquisition method


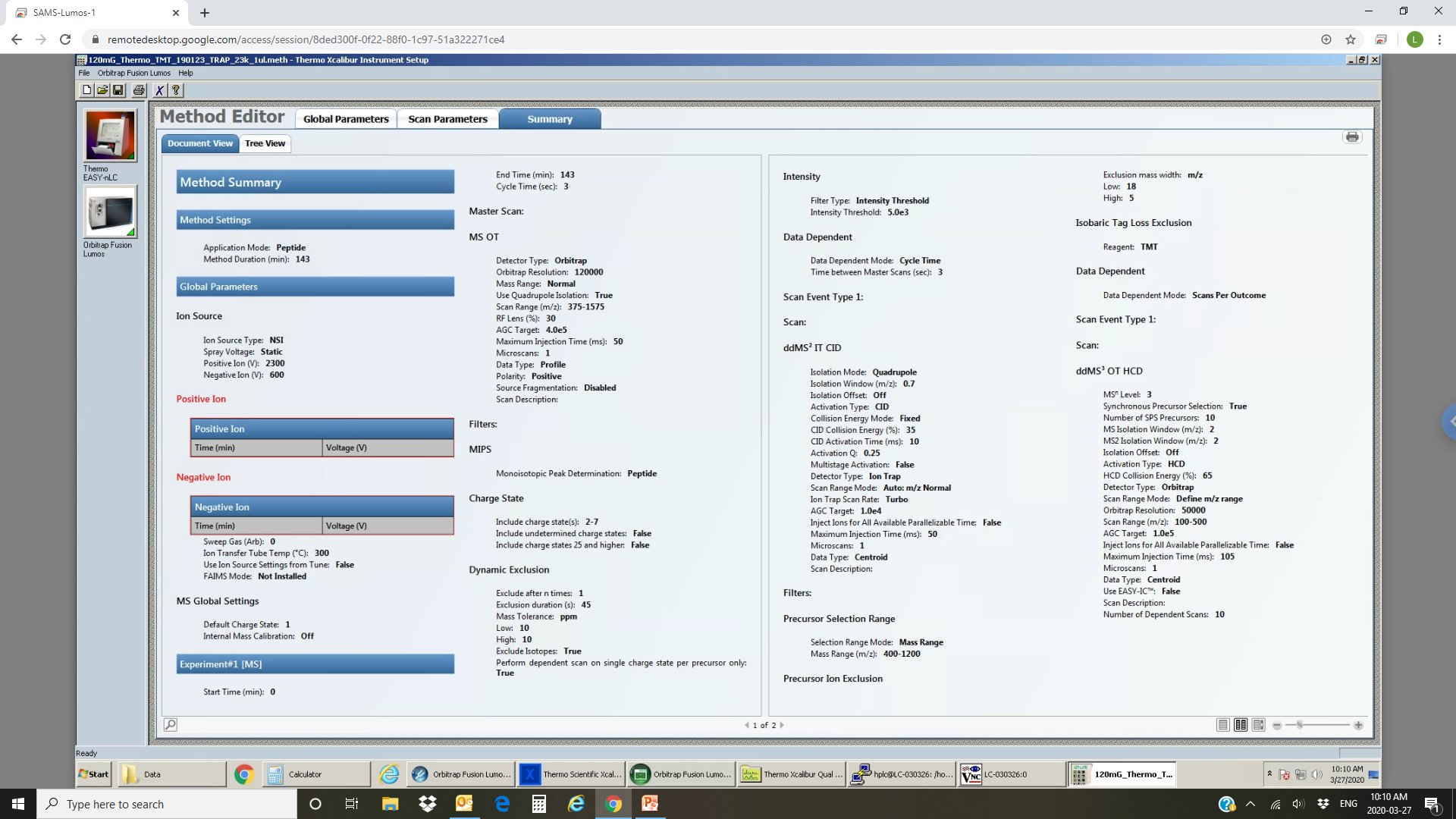


Figure S3 Summary of the TMT MS3 acquisition method


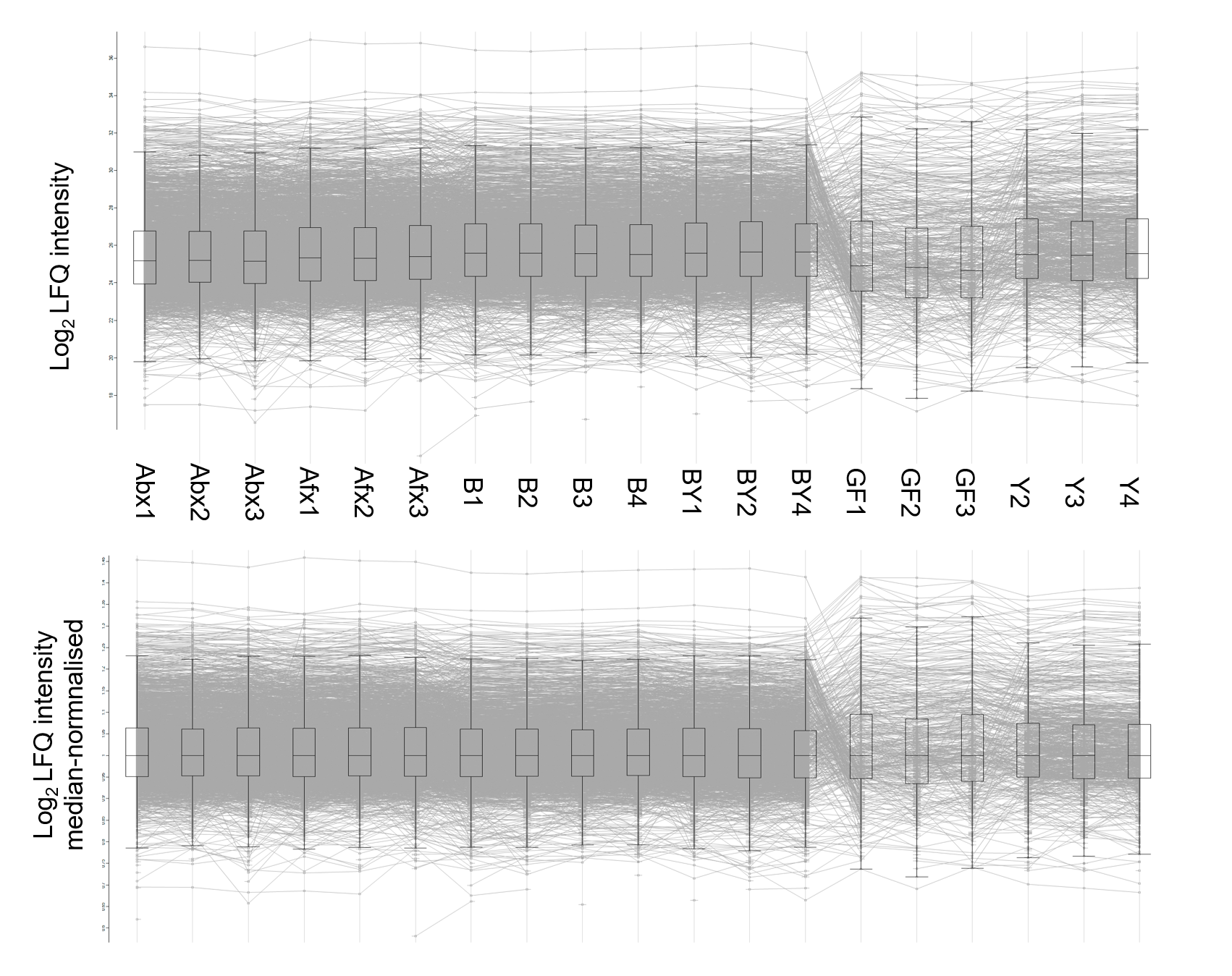


**Figure S4** Normalisation by median decreased variability introduced during sample injection. The upper panel shows LFQ intensity data before normalisation, while the lower panel shows the same data after median normalisation.


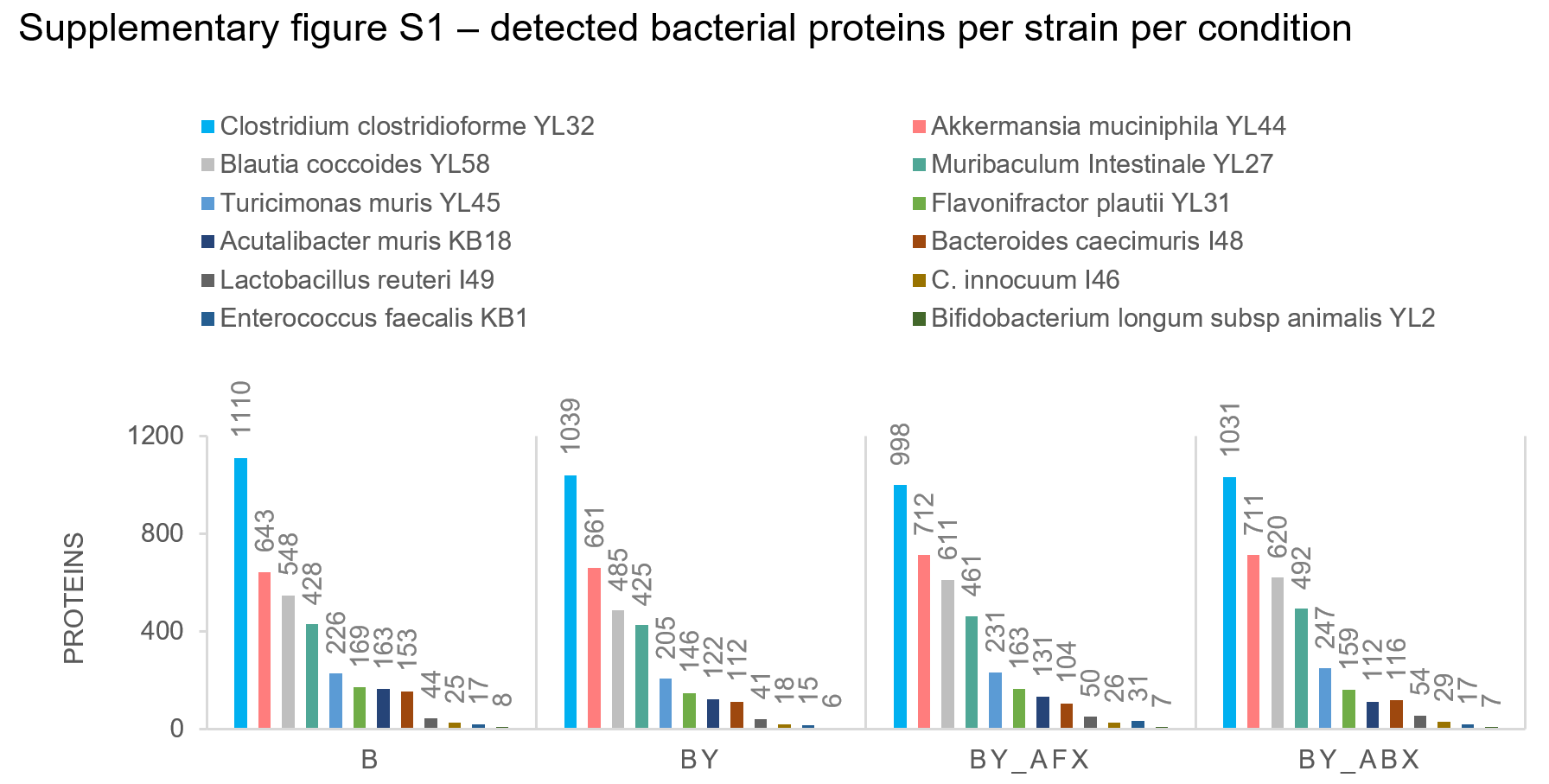


**Figure S5**. Detected bacterial proteins per strain per condition. Abbreviations of the mice treatment groups: B, bacteria; BY, bacteria-yeast; BY_ABX/AFX, bacteria-yeast, and antibiotic or antifungal treatment.


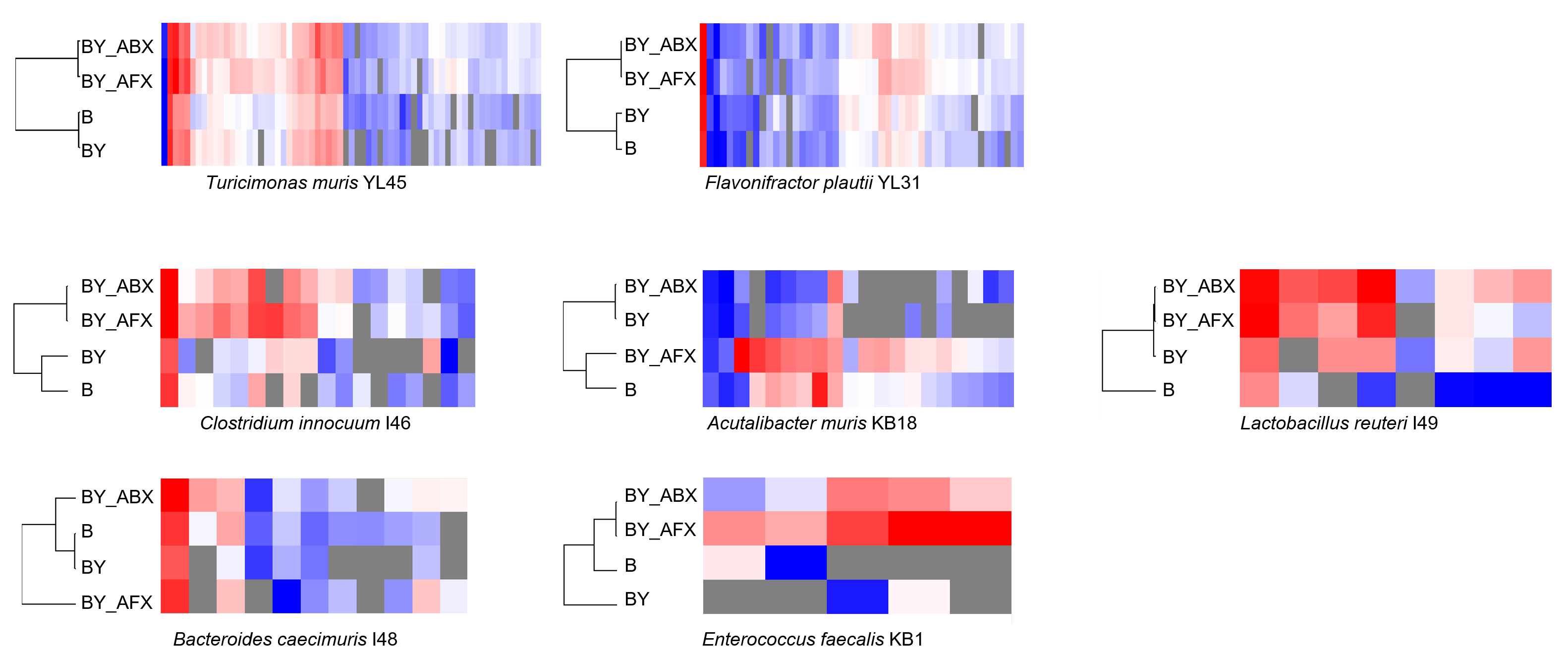


**Figure S6** Bacterial proteomes response to antimicrobials and the presence of fungi – seven strains with a low number of quantified proteins. Abbreviations of the mice treatment groups: B, bacteria; BY, bacteria-yeast; BY_ABX/AFX, bacteria-yeast, and antibiotic or antifungal treatment.


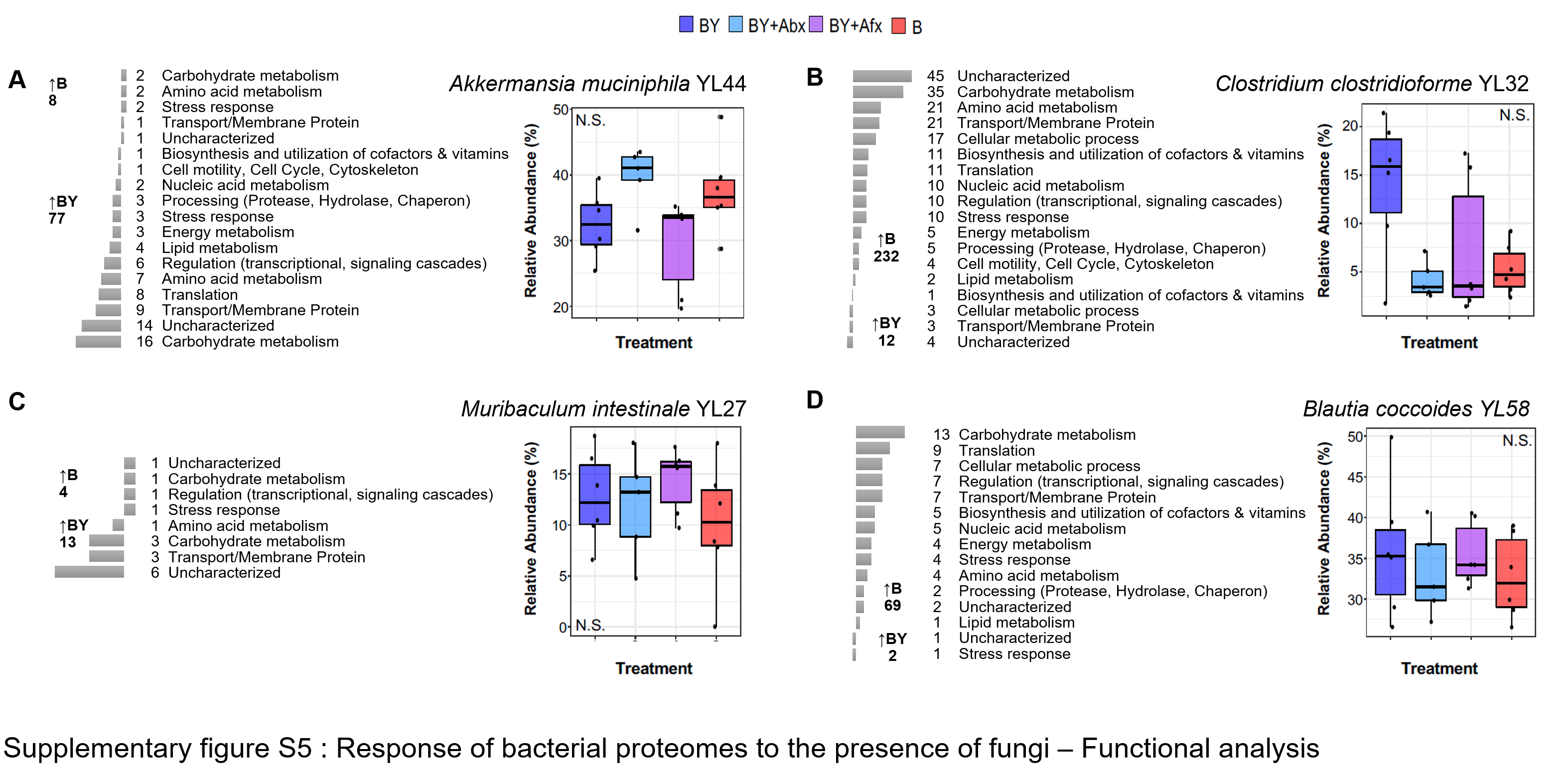


**Figure S7**. Number and functional classes of differentially detected bacterial proteins in either B or BY mice groups for A) *A. muciniphila* YL44, B) *C. clostridioforme* YL32, C) *M. intestinale* YL27, and D) *B. coccoides* YL58. Protein functional annotation was downloaded from the UniProtKB database and compared to annotations obtained using the DAVID and STRING-db tools. The relative abundance of the strains based on 16s rRNA sequencing data is shown on each panel’s right. Number of proteins in each functional category is indicated next to the bar plots (scale not comparable between A-D). N.S. – not significant. Abbreviations of the mice treatment groups: B, bacteria; BY, bacteria + fungi; BY+ABX/AFX, bacteria + fungi and antibiotic or antifungal treatment; GF, germ-free; Y, fungi/yeast.

**
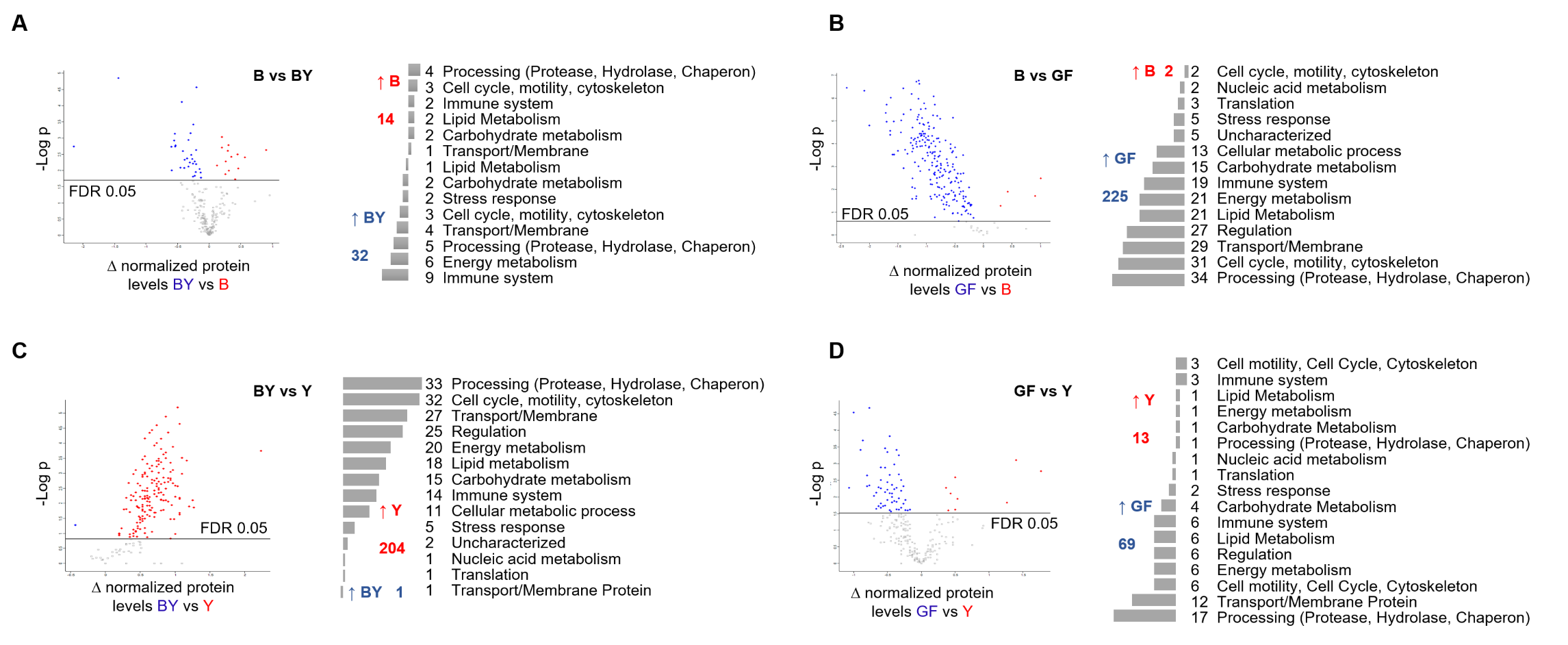
****Figure S8**. The response of mouse fecal proteome to the presence of microbial consortiums containing fungi and/or bacteria. Statistical comparison of quantified mouse proteins (t-test, FDR 5%) was performed in the Perseus proteomic software on A) B and BY, B) B and GF, C) BY and Y, and D) GF and Y groups. Functional classes were based on the mouse protein annotations derived from the UniProtKB database and compared to those obtained by the DAVID and STRING-db tools. Abbreviations of the mice treatment groups: B, bacteria; BY, bacteria + fungi; GF, germ-free; Y, fungi/yeast.

**Table S8** Number of proteins with significantly increased levels (t-test, FDR 5%) for selected bacterial strains and between different mice groups. Abbreviations of the mice treatment groups: B, bacteria; BY, bacteria + fungi; BY_ABX/AFX, bacteria + fungi and antibiotic or antifungal treatment; GF, germ-free; Y, fungi/yeast.

| Bacterial Strain | Proteins Quantified | ANOVA FDR 5% | THSD | B vs BY | | BY_ABX vs BY_AFX | | BY vs BY_AFX | | BY vs BY_ABX | | B vs BY_AFX | | B vs BY_ABX | |
| --- | --- | --- | --- | --- | --- | --- | --- | --- | --- | --- | --- | --- | --- | --- | --- |
|  |  |  |  | B | BY | ABX | AFX | BY | AFX | BY | ABX | B | AFX | B | ABX |
| *Akkermansia muciniphila YL44* | 570 | 308 | 280 | 5 | **67** | 18 | 17 | 4 | **105** | 4 | **114** | 3 | **204** | 1 | **206** |
| *Muribaculum Intestinale YL27* | 393 | 233 | 206 | 6 | **20** | **48** | 15 | 7 | **98** | 0 | **122** | 22 | **109** | 4 | **179** |
| *Blautia coccoides YL58* | 502 | 271 | 244 | **51** | 1 | **44** | 13 | 5 | **142** | 4 | **157** | 7 | **156** | 10 | **127** |
| *Clostridium clostridioforme YL32* | 921 | 553 | 456 | **100** | 7 | **125** | 27 | **104** | 83 | 36 | **147** | **211** | 80 | **120** | 96 |
